# DMP-UNet: a multimodal fusion network for high-precision nitrogen status monitoring in wheat from UAV multispectral images

**DOI:** 10.3389/fpls.2026.1909856

**Published:** 2026-09-03

**Authors:** Qingqing Hong, Siqi Cao, Bohan Hu, Changwei Tan, Zhenghua Zhang, Bin Li, Hongwei Zhang

**Affiliations:** 1 College of Information and Artificial Intelligence, Yangzhou University, Yangzhou, China; 2 Joint International Research Laboratory of Agriculture and Agri-Product Safety of the Ministry of Education of China, Yangzhou University, Yangzhou, China; 3 Jiangsu Key Laboratory of Crop Genetics and Physiology/Jiangsu Key Laboratory of Crop Cultivation and Physiology/Jiangsu Co-Innovation Center for Modern Production Technology of Grain Crops, Agricultural College of Yangzhou University, Yangzhou, China

**Keywords:** deep learning, image segmentation, multimodal feature fusion, UAV imagery, wheat nitrogen segmentation

## Abstract

Achieving high-precision monitoring of wheat nitrogen levels is essential for boosting yield and grain quality, yet the remote sensing community has largely confined itself to single-modal data sources, missing the synergistic benefits of multimodal fusion. To address this, we propose a multimodal feature fusion network that simultaneously utilizes RGB, multispectral, and vegetation index data. The network employs dual-branch encoders to extract modality‑specific features from fused images composed of NDVI, green, and near‑infrared bands; a deformable bidirectional cross‑attention module aligns pixel‑level features and enhances cross‑modal interactions; a modality‑aware pyramid attention feature pyramid network fuses semantic information across scales and modalities; and a dual‑path decoder separates background from wheat regions, with a foreground decoder guided by vegetation attention maps to classify nitrogen status into four levels: Enrichment, Optimum Level, Moderate Nitrogen Deficiency, and Severe Nitrogen Deficiency. Experimental results demonstrate that our method outperforms state‑of‑the‑art models such as DeepLabV3+, U‑Net, U‑Net++, and Attention UNet. Modality contribution analysis confirms the complementary roles of RGB and MS, while ablation studies validate the effectiveness of each key module. These findings confirm the practicality and efficiency of the proposed approach for precise nitrogen monitoring in wheat, highlighting its potential for optimizing fertilization strategies in precision agriculture.

## 1 Introduction

Wheat ranks as one of the predominant staple crops globally, playing a vital role in ensuring food security (Shiferaw et al., 2013; Li J. et al., 2025). The availability of nitrogen is pivotal in regulating the growth, photosynthetic efficiency, and yield formation of wheat (Javed et al., 2022). However, the excessive application of nitrogen can lead to inefficiencies in resource use (Good and Beatty, 2011), environmental pollution, and potential health hazards (Gu et al., 2023). On the other hand, a deficiency in nitrogen impedes chlorophyll synthesis, leading to leaf chlorosis, stunted growth, and, in severe instances, yellow dwarf disease (Banks et al., 1995). Such deficiencies ultimately compromise the quality and yield of the grain. Consequently, precise and timely monitoring of the nitrogen status in crops is crucial for guiding precision fertilization and enhancing the sustainability of agricultural practices (Mulla, 2013).

Traditionally, nitrogen monitoring in fields has depended on manual sampling and subsequent laboratory analysis. These methods are laborious, time-consuming, destructive, and not suited for large-scale or real-time applications (Zheng et al., 2018). In recent years, the use of unmanned aerial vehicles (UAVs) for remote sensing has gained traction due to their operational flexibility, rapid data acquisition, cost-efficiency, and ability to carry multiple sensors (Yang T. et al., 2025; Bagheri and Kafashan, 2025; Jiang et al., 2025). UAVs provide a versatile and effective method for capturing field imagery with high spatial resolution and extensive coverage, thus offering a valuable tool for monitoring wheat growth and nitrogen status (Fu et al., 2022).

Researchers have generally developed spectral or vegetation index features and integrated them with machine learning regression models such as partial least squares regression (PLSR), support vector regression (SVR), and least absolute shrinkage and selection operator (LASSO) to estimate nitrogen levels (Yang et al., 2019).

**Figure 1:**
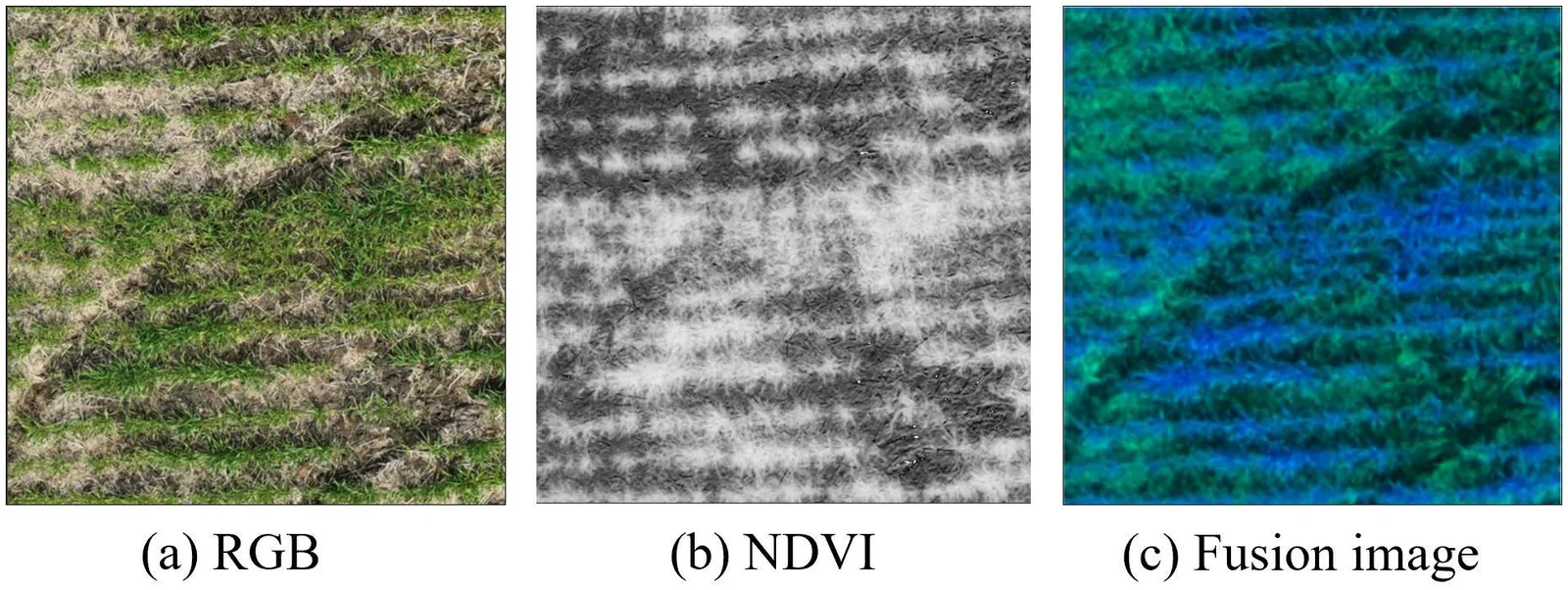
Examples of multimodal data. **(a)** RGB image; **(b)** NDVI map; **(c)** multispectral fusion image derived from NDVI, Green, and NIR.

For example, one study explored the feasibility of estimating plant nitrogen content using UAV multispectral (MS) images (Yang M. D. et al., 2025). Furthermore, the integration of high-overlap multiview UAV images with the SVR algorithm has significantly enhanced the accuracy of estimating nitrogen concentration in the leaves and plants of winter wheat, offering a cost-effective approach for agricultural remote sensing monitoring (Lu et al., 2022). Nonetheless, these methodologies are often limited by their reliance on handcrafted features and their struggles to capture nonlinear and multiscale relationships, as well as their inefficacy in integrating multimodal data such as RGB and MS imagery. As illustrated in **Figure 1**, RGB imagery clearly captures canopy structure, leaf morphology, and spatial texture, which are beneficial for delineating plant boundaries. In contrast, the NDVI map highlights vegetation vigor and spectral variations associated with nitrogen status, while the MS fusion image derived from NDVI, Green, and NIR further emphasizes nitrogensensitive spectral responses. These complementary characteristics motivate the integration of RGB and multispectral data for nitrogen level segmentation.

The application of deep learning has shown significant potential for crop monitoring. Convolutional neural networks (CNNs), particularly the U-Net architecture proposed by (Ronneberger et al., 2015), has been extensively utilized for image segmentation on modern GPUs. For example (Wang et al., 2023), developed a UAV-based multispectral model for pixel-level prediction of tea plant nitrogen content, while (Zhang et al., 2020) used a U-Net framework with UAV RGB imagery to segment nitrogen stress in purple rapeseed leaves. In addition, the evaluation of multiple U-Net variants for nitrogen segmentation in grain fields using RGB, near-infrared (NIR), and normalized difference vegetation index (NDVI) data reported by (Blekanov et al., 2023) further demonstrated the effectiveness of multimodal inputs. Zhao et al. (2025a) proposed a Mamba-based framework integrating super-resolution reconstruction with semantic segmentation for UAV-assisted blueberry maturity monitoring. Tao et al. (2025) developed a deep learning framework combining super-resolution reconstruction and semantic segmentation for automatic segmentation of weeds and crops in tobacco fields using UAV imagery. In the domain of object detection, several lightweight YOLO-based models have been proposed for specific agricultural targets, including FP-YOLO for UAV-based small object detection (Xie et al., 2026), Mussel-YOLO for freshwater mussel detection using acoustic video cameras (Zhao et al., 2025b), Succulent-YOLO for succulent farmland monitoring (Li H. et al., 2025), Rose-Mamba-YOLO for greenhouse rose detection (Fan and Chai, 2026), and Rose-YOLO for rose growth monitoring (Shi et al., 2026).

Nevertheless, distinct differences exist between prior investigations and the current work. Most published approaches prioritize object detection over semantic segmentation, excluding limited studies on blueberry maturity evaluation and weed–crop segmentation. Corresponding outputs are produced as bounding boxes, rather than precise pixel-level classification maps. Existing research is largely focused on fruit maturity assessment, weed–crop discrimination and ornamental plant monitoring, while the present study targets wheat nitrogen nutrition status, a critical attribute that directly governs crop yield and grain quality. Technically, conventional methods predominantly rely on single-modal RGB imagery combined with super-resolution processing or detection-oriented optimization strategies. In comparison, the proposed DMP-UNet is established for multimodal feature fusion, through which RGB data and NDVI, Green and NIR multispectral information are comprehensively integrated via cross-modal attention mechanisms. The developed model enables fine-grained pixel-level classification to categorize wheat nitrogen status into four grades: Severe Deficiency, Moderate Deficiency, Optimum and Enrichment. This classification task presents inherent challenges due to the subtle spectral similarities between adjacent nitrogen levels and the continuous spatial variation of crop nitrogen status in actual field environments.

Despite the progress achieved by existing agricultural monitoring methods, the complementary advantages of multimodal data are still underutilized. Most existing models simply implement feature concatenation for RGB and multispectral inputs, and explicit interaction modeling and heterogeneity adaptation between different modalities are neglected. Since RGB images capture structural and texture details whereas multispectral data provide nitrogen-sensitive spectral features and vegetation indices, effective multimodal fusion is regarded as essential for reliable and accurate nitrogen status evaluation. To mitigate this limitation, a UAV imagery-based multimodal feature fusion segmentation network is developed in this study. Cross-modal feature correlations are explicitly modeled and complementary information from dual-source imagery is fully integrated to facilitate accurate pixel-level monitoring of wheat leaf nitrogen levels. The main contributions of this study are summarized as follows:

- A dual-branch encoder was engineered to extract complementary structural and spectral features, where the RGB branch captured plant texture and morphology, and the MS branch concentrated on nitrogen-sensitive spectral information.
- A deformable bidirectional cross-attention module (DBCAM) was integrated to align multimodal features and augment cross-modal fusion.
- A modality-aware pyramid attention (MAPA) module, in tandem with a feature pyramid network (FPN), was introduced to amplify nitrogen-sensitive representations across various scales while maintaining detailed leaf and canopy structures.
- A dual-path decoder was utilized, wherein a background branch generates vegetation attention maps to direct the foreground branch, effectively minimizing background interference.

The remainder of this paper is organized as follows. Section 2 introduces the study area, UAV data acquisition, and dataset preparation, and presents the proposed DMP-UNet architecture and multimodal feature fusion strategy. Section 3 describes the experimental setup and reports the evaluation results and analyses. Finally, Section 4 summarizes the proposed approach of this study and outlines directions for future research.

## 2 Materials and methods

### 2.1 Field experiment

**Figure 2:**
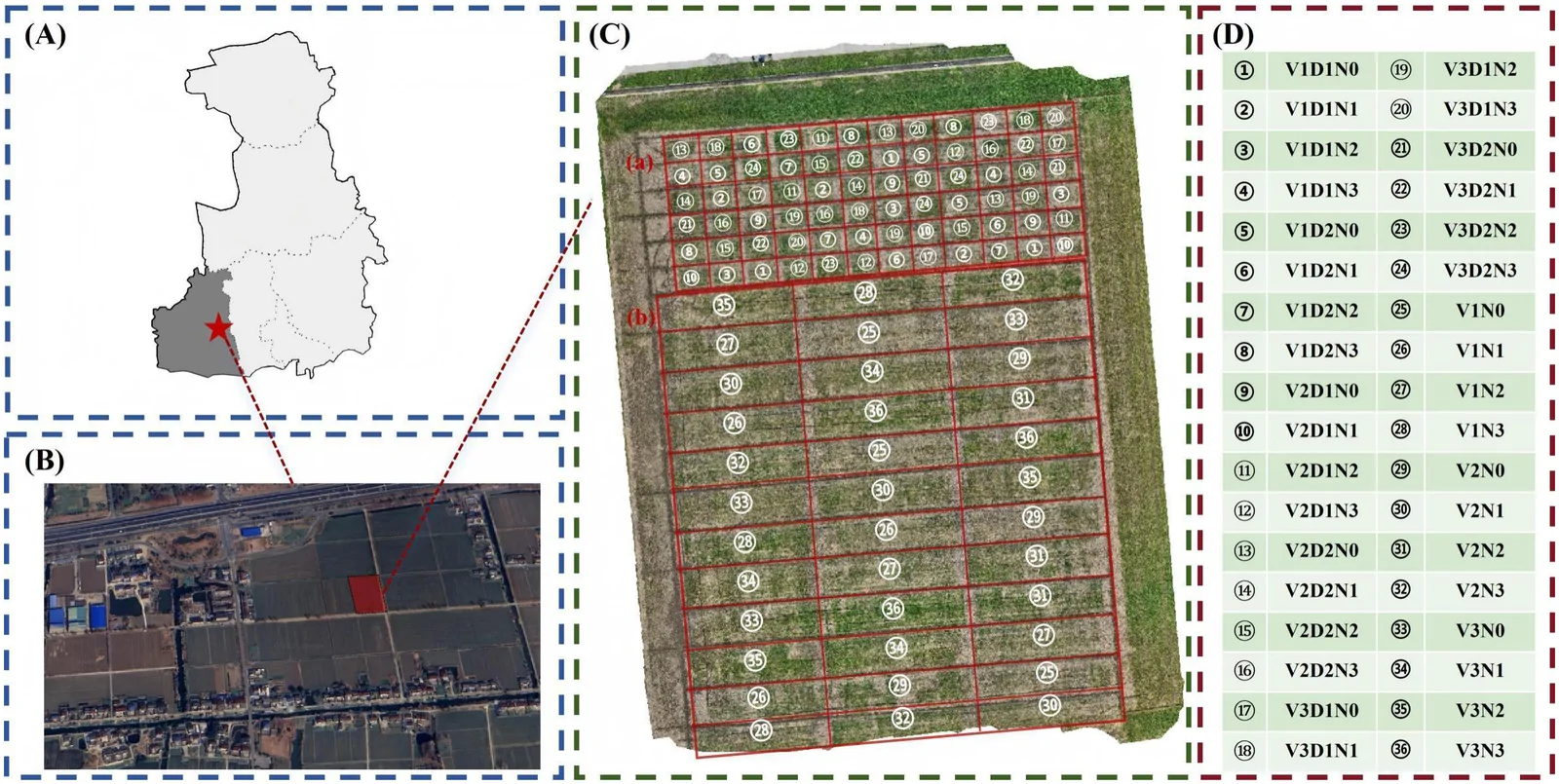
**(A)** The geographic location of the experimental fields in Yizheng City, Yangzhou, Jiangsu Province. **(B)** The Xinjizhen experimental site. **(C)** The experimental fields, comprising two areas: **(a)** with 72 plots and **(b)** with 36 plots, where different plot numbers indicate different treatment methods. **(D)** Treatment methods corresponding to the plot numbers.

The experimental field utilized for this study is situated in Xinji Town, Yizheng City, within the Yangzhou region of Jiangsu Province, China (32◦19′12′′N, 119◦18′36′′E), as depicted in **Figure 2**. Located in the lower reaches of the Yangtze River, the site is characterized by a typical subtropical monsoon climate with distinct seasonal variations. The area receives an average annual precipitation of 1037.4 mm and maintains a mean annual temperature of 15.4◦*C*, accompanied by approximately 2140 hours of sunshine per year. The region’s fertile soil and deep soil strata create optimal conditions for wheat cultivation. For the purposes of this experiment, three wheat cultivars were chosen: V1 (“Yangfu Mai 21”), V2 (“Yangfu Mai 13”), and V3 (“Yang Mai 28”). Each variety has a growth period of about 210 days and demonstrates robust resistance to prevalent wheat diseases, which facilitates the smooth conduct of the experiment. The study involved a field experiment with different nitrogen fertilizer gradients. Four nitrogen treatments were administered: N0 (0 kg·hm−^2^), N1 (50 kg·hm−^2^), N2 (225 kg·hm−^2^), and N3 (300 kg·hm−^2^). Phosphorus and potassium were applied as basal fertilizers in accordance with standard agricultural practices, while nitrogen applications were split between basal and heading-stage applications. The experimental field was divided into two zones (**Figure 2C**).

- Zone A included 72 plots, each measuring 3m × 4m, organized into three replicates across four nitrogen rates, three cultivars, and two planting densities: D1 (150 × 10^4^ plants· hm−^2^) and D2 (225 × 104 plants·hm−2).
- Zone B encompassed 36 plots, each 5m × 20m, arranged in three replicates for the same nitrogen levels and cultivars, but with a uniform planting density of 225 × 10^4^ plants·hm−^2^.

This design was intended to simulate a range of nitrogen environments, thereby ensuring adequate data variability and establishing a distinct nitrogen gradient for subsequent analysis. All plots were subject to identical irrigation and field management practices, adhering to uniform agronomic standards to guarantee the consistency of the experiment and the reliability of the data collected.

### 2.2 Data acquisition

The UAV imagery utilized in this study was captured using a DJI Mavic 3 MS UAV, as detailed in **Table 1**. his platform is outfitted with an RGB camera and an MS sensor, which includes four spectral bands: Green (560 ± 16 nm), Red (650 ± 16 nm), Red-Edge (730 ± 16 nm), and NIR (860 ± 26 nm). This configuration facilitates the high-quality acquisition of imagery of the wheat canopy, essential for subsequent analyses.

**Table 1:** Specifications of UAV and camera.

Bare metal weight	951 g
Maximum flight time	43 minutes
Maximum range	32 km
Maximum photo size	2592×1944
RGB image sensor	4/3 CMOS, pixels 20 million
RGB photo format	JPEG/DNG (RAW)
MS image sensor	1/2.8-inch CMOS with 5 million pixels
MS photo format	TIFF
MS camera band	Green (G): 560nm ± 16nm Red (R): 650nm ± 16nm

The flight route was meticulously planned to encompass the boundaries of the experimental field. The UAV’s sensor was aligned to point directly downward, and the flight altitude was maintained at 12 m with a speed of 1 m/s. Images were captured automatically at each waypoint, with a heading overlap of 80% and a side overlap of 70% (Zhang et al., 2020), to guarantee comprehensive coverage of the experimental field during the flight mission.

Concurrent with the UAV mission, manual sampling was conducted in two distinct regions of the experimental field. Representative wheat plants exhibiting uniform growth were selected from each of the 72 plots in Area (a) and 36 plots in Area (b), clipped, and stored in labeled sample bags with unique identifiers. The geographic coordinates of each sampling site were documented using a portable GPS device, facilitating the spatial alignment of UAV imagery with ground sampling data.

Following the mission, the raw UAV imagery from the experimental field was uploaded into DJI Terra for stitching and reconstruction. The resultant data included an RGB image, alongside MS image data across the four bands: R, G, NIR, and RE. The DJI Mavic 3 Multispectral UAV is equipped with an onboard sunlight sensor that records incident irradiance during flight, and DJI Terra automatically performs illumination compensation and converts raw digital numbers (DN) to reflectance values using these recorded data. The multispectral camera array and RGB camera share fixed calibration parameters, and DJI Terra applies these intrinsic and extrinsic calibration parameters to accomplish geometric band-to-band registration and resampling during orthomosaic generation. The flight mission was designed with 80% forward overlap and 70% side overlap to ensure sufficient tie-point matching and reconstruction accuracy, which is a standard configuration for producing high-quality orthomosaics.

The harvested wheat plants were initially washed, and their roots detached. The samples were then blanched in an oven at 105◦C for 30 minutes before the temperature was reduced to 80◦C, allowing the samples to dry to a constant weight (Blekanov et al., 2023). The dry biomass of each sample was recorded, and the per-hectare dry biomass of the wheat was calculated (Zhai et al., 2025). Subsequently, all plant organs were ground, and their nitrogen content was analyzed using a Kjeltec*™* 8400 fully automatic Kjeldahl nitrogen analyzer.

### 2.3 Dataset construction

The NDVI was utilized to characterize the physiological status of vegetation, functioning as a pivotal indicator for assessing nitrogen levels in wheat (Fan et al., 2024). The mean reflectance values for each spectral band were obtained from MS imagery at the GPS sampling points. The NDVI was subsequently calculated using the R and NIR spectral bands according to the following equation:

(1) $$NDVI=\frac{NIR-R}{NIR+R}$$

where NIR and R represent the reflectance of the NIR and Red bands, respectively.

The G band, which is located within the chlorophyll reflection peak region, exhibits high sensitivity to chlorophyll variations and is thus closely correlated with nitrogen concentration (Gitelson et al., 2003). The NIR band is predominantly affected by leaf and canopy structure, providing essential information on biomass and canopy morphology (Gitelson and Merzlyak, 1998). Together, these spectral and vegetation index features establish a robust foundation for spatial modeling and classification of wheat nitrogen levels across the study area.

Wheat nitrogen nutrition levels were classified into four grades, and corresponding image label maps were generated. This classification leveraged the critical nitrogen dilution curve theory by (Justes et al., 1994), which elucidates a negative relationship between aboveground dry matter (W) and nitrogen concentration (Nc). This theory provides a quantitative basis for nitrogen diagnosis. The Nitrogen Nutrition Index (NNI) (Lemaire et al., 2008; Li et al., 2022), derived from this theory, quantifies the equilibrium between nitrogen supply and biomass accumulation, serving as an essential indicator for evaluating the nitrogen status in wheat.

In this study, the critical nitrogen concentration model for wheat was employed as given in Equation 2:

(2) $$N_{c}=4.16\times W^{-0.41}$$

where *N_c_*represents the critical nitrogen concentration (%), and *W* denotes the aboveground dry biomass. The NNI was calculated using the formulation given in Equation 3:

(3) $$NNI=\frac{N_{act}}{N_{c}}$$

where *N_act_*is the measured nitrogen concentration (%) of the plant sample. An *NNI* value of 1 indicates optimal nitrogen nutrition, whereas values of *NNI <* 1 and *NNI >* 1 correspond to nitrogen deficiency and excess, respectively.

Previous studies (Huangfu et al., 2025; Lemaire et al., 2008; Bo et al., 2021) have documented that wheat maintains optimal nitrogen status when *NNI* ranges from 0.95 to 1.05. Values below 0.95 signify nitrogen limitation, while those above 1.05 indicate an excessive nitrogen supply, which could lead to potential nitrogen loss or environmental pollution.

Considering the continuous variation and nonlinear physiological response of wheat to nitrogen levels, *NNI* values were segmented into four categories to facilitate refined nitrogen classification. Additionally, a background category was introduced to exclude non-wheat or invalid regions, thus enhancing the precision of nitrogen segmentation within the deep learning model (**Table 2**). In this study, the construction of label maps was achieved using the Random Forest (RF) supervised classification model (Belgiu and Dragut, 2016), which incorporated field-measured nitrogen content along with NDVI, Green, and NIR band reflectance. The RF algorithm is recognized for its robust non-linear modeling capabilities and its resilience to noise, especially in scenarios characterized by small sample sizes. Additionally, it effectively quantifies the importance of features, thereby facilitating stable and efficient mapping between nitrogen-related attributes and measured data. Spatial matching involved pairing the measured nitrogen content at each sampling point with the spectral characteristics of the corresponding pixels to create a training dataset. Subsequently, the RF classifier was trained using this dataset. Through the established RF mapping relationship, point-based observations were extrapolated across the entire image, resulting in a comprehensive pixel-level nitrogen label map. These maps were stored in GeoTIFF format with spatial reference coordinates aligned with the original remote sensing imagery, providing a visual representation of the nitrogen distribution across the experimental field and serving as reliable supervisory data for subsequent deep segmentation models.

**Table 2:** Definition of a label.

Class	Nitrogen level	NNI
0	Nitrogen Enrichment	NNI > 1.05
1	Optimum Level	0.95≤ NNI ≤1.05
2	Moderate Nitrogen Deficiency	0.85≤ NNI <0.95
3	Severe Nitrogen Deficiency	NNI <0.85
4	Background	–

To leverage complementary information from different image types, RGB and NDVI-Green-NIR MS fusion images were processed as distinct inputs. The RGB images captured high-resolution texture and spatial structure, preserving detailed leaf contours and spatial nuances. Conversely, the MS fusion images, by integrating NDVI, Green, and NIR bands, were able to capture the physiological status of vegetation, chlorophyll content, and canopy structure, thus enhancing spectral sensitivity to nitrogen. This dual-input strategy enabled the model to effectively integrate complementary spectral and spatial features.

**Figure 3:**
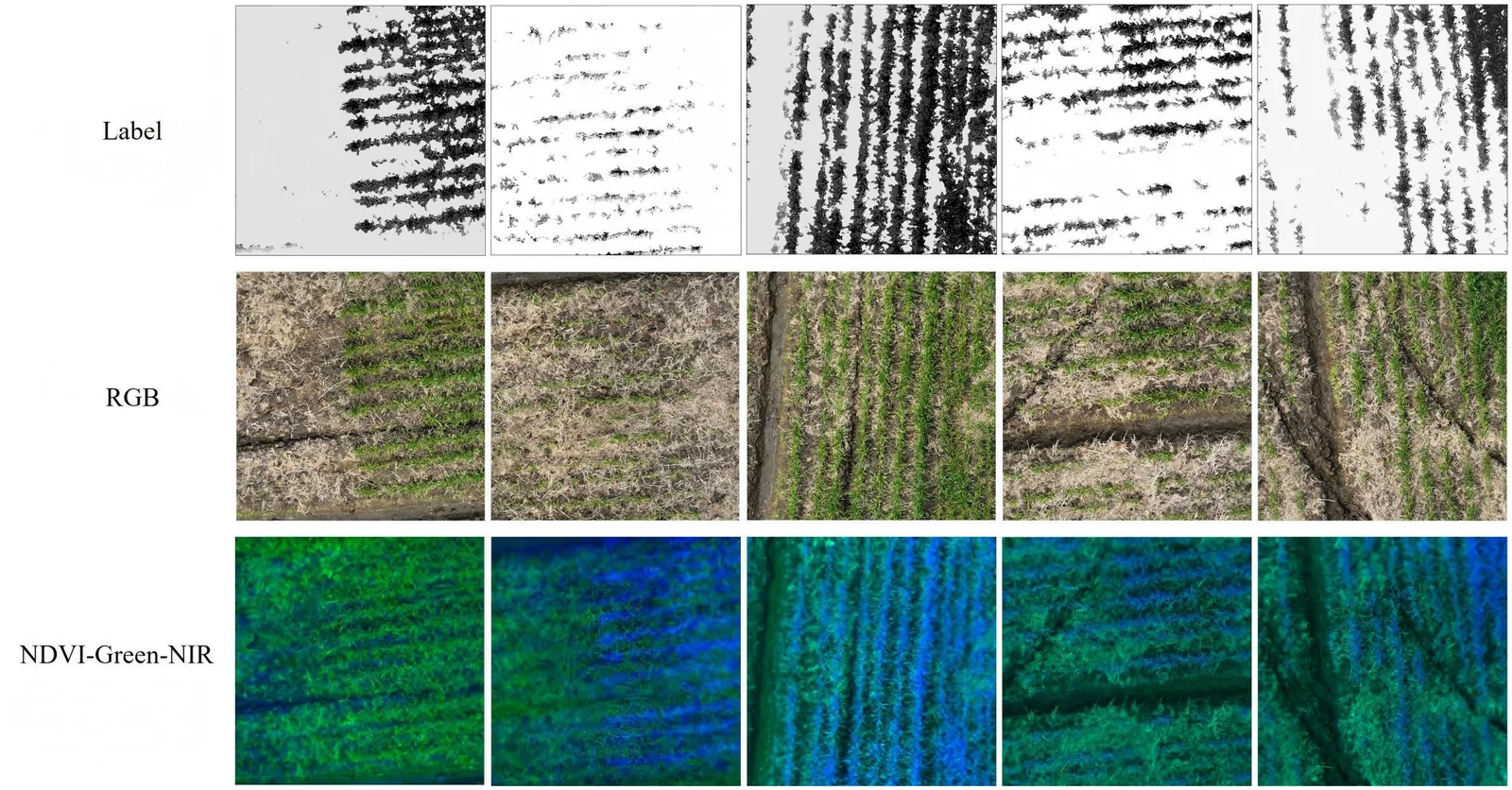
Example of the dataset. Each sample consists of an RGB image, an NDVI–Green–NIR multispectral fusion image, and a corresponding label map.

Accurate spatial alignment between input images and label maps was achieved by uniformly cropping RGB, NDVI-Green-NIR, and label images to dimensions of 5280 × 3956 pixels. These were further segmented into blocks of 512 × 512 pixels using sliding windows and random cropping techniques. This method balanced memory usage and computational efficiency while preserving the local field structure and information on nitrogen distribution. Pixel values in each label map were strictly aligned with defined nitrogen levels to ensure precise one-to-one mapping with the original images. To enhance model generalization and increase data diversity, data augmentation techniques were applied, including random rotation, horizontal flipping, and brightness adjustment. After cropping and augmentation, a total of 3,420 image samples were produced, encompassing a variety of scenes with different nitrogen levels and image characteristics. The dataset was divided into training (70%), validation (20%), and test (10%) sets, with each sample consisting of an RGB image, an NDVI-Green-NIR image, and a corresponding label map (**Figure 3**), thereby providing high-quality inputs for training the multimodal semantic segmentation model.

### 2.4 Overview of method

**Figure 4:**
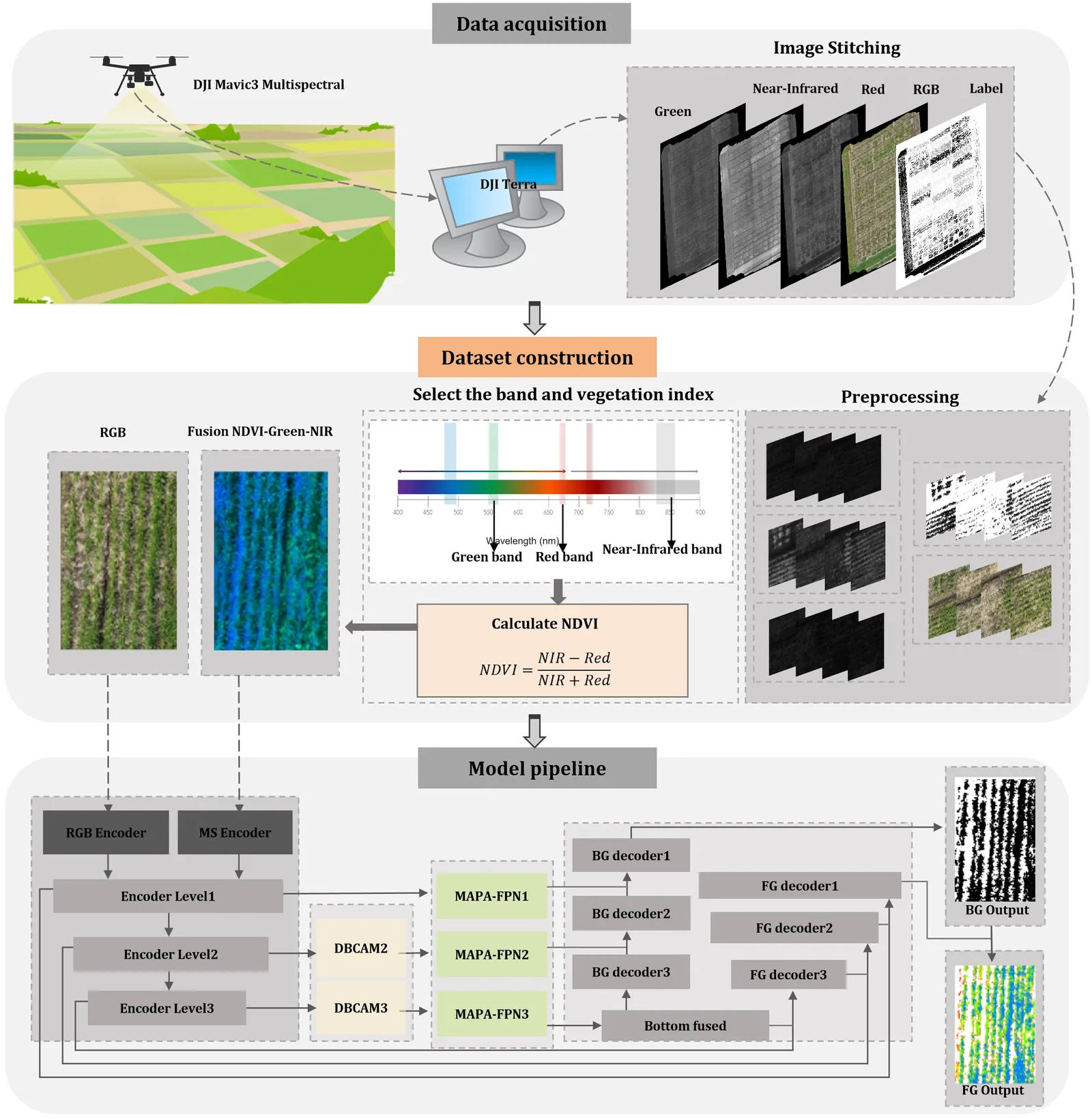
Pipeline scheme for the overall process of nitrogen level segmentation.

The technical workflow of this study is delineated in **Figure 4** and encompasses three primary components: data acquisition, dataset construction, and model pipeline. MS and RGB imagery, spanning R, G, NIR, and RGB bands, were captured using a DJI Mavic 3 MS UAV. The images were processed in DJI Terra to produce orthorectified plots, while corresponding labels were generated. Select bands were utilized to compute the NDVI, and fusion images combining RGB, NDVI, G, and NIR were created, cropped, and standardized to ensure uniform samples. Subsequently, a multimodal adaptive feature fusion network was developed. Dual encoders were employed to extract textural-morphological and spectral features, which were aligned and fused through a DBCAM. A MAPA-FPN was implemented to enhance contextual representation and facilitate interaction across multiple scales. Ultimately, a vegetation-attention-guided dual decoder performed the segmentation of nitrogen levels, enabling precise identification of wheat nitrogen status under complex field conditions.

### 2.5 DMP-UNet network

The DMP-UNet is a multimodal image segmentation architecture designed to integrate complementary information from both RGB and NDVI-Green-NIR fusion MS images (subsequently referred to as MS images). This architecture incorporates two parallel encoder branches that independently extract hierarchical features from each image modality, thereby capturing both spatial details and spectral information. These features are then merged through a series of multimodal fusion blocks that effectively combine cross-modal information while preserving the characteristics specific to each modality. Skip connections are employed between the encoder and decoder layers to facilitate multiscale feature propagation and enhance precise localization.

**Figure 5:**
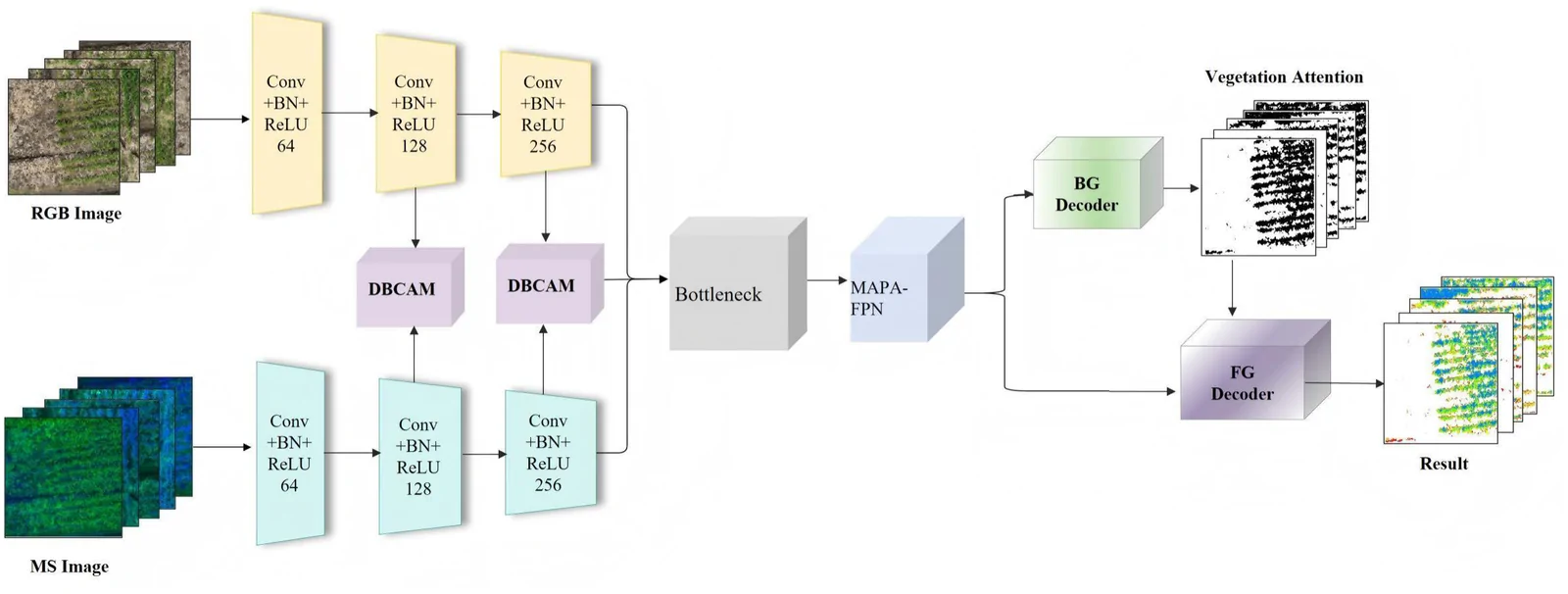
Schematic diagram of the DMP-UNet architecture.

The decoder progressively reconstructs high-resolution segmentation maps by upsampling and amalgamating fused features at various scales. Additionally, attention mechanisms are utilized to highlight informative features, augment cross-modal interactions, and minimize redundant information. Ultimately, the network outputs pixel-level segmentation maps that provide detailed classification of nitrogen levels or other specified categories within the field (**Figure 5**).

#### 2.5.1 Deformable bidirectional cross-attention module

**Figure 6:**
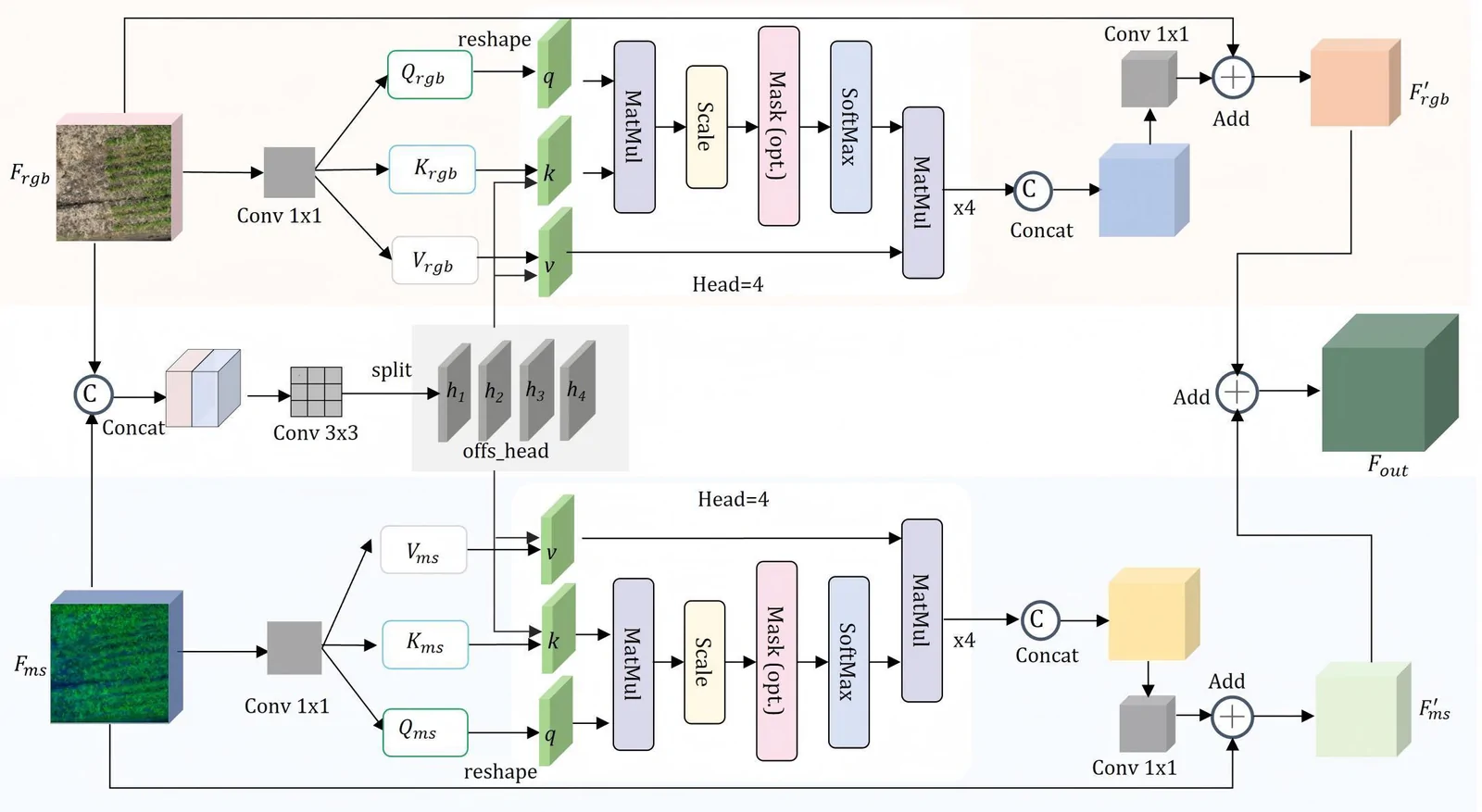
Structure of the DBCAM.

RGB images provide high-resolution texture and color information, but they correlate weakly with nitrogen content. Conversely, MS images capture nitrogen-related physiological features, such as vegetation indices and spectral reflectance, but often lack spatial detail and sharp edges. To exploit the complementary strengths of these modalities and address issues of spatial misalignment and scale differences, a DBCAM was developed (**Figure 6**). This module utilizes dynamic sampling attention to capture long-range dependencies, suppress irrelevant information, and facilitate bidirectional enhancement for adaptive cross-modal feature fusion.

Specifically, the RGB feature maps *F_rgb_*and MS feature maps *F_ms_*are first projected into low-dimensional representations for attention computation.This is achieved by using separate 1×1 convolutions to generate Query (Q), Key (K), and Value (V) vectors for each modality, denoted as $Q_{rgb}$, $K_{rgb}$, $V_{rgb}$ and $Q_{ms}$, $K_{ms}$, $V_{ms}$. Simultaneously, the two modal features are concatenated along the channel dimension and fed into a 3×3 convolutional layer. The kernels of this layer aggregate cross-modal contextual information within a local receptive field and output deformable offsets Δ*p* for each attention head. These offsets enable the network to learn geometric misalignments between the two modalities and produce distinct sampling patterns for each head. The Δ*p* are generated according to Equation 4 as follows:

(4) $$\text{Δ}p=f_{offset}(\text{Concat}(F_{rgb},\;F_{ms}))\in {\mathbb{R}}^{H\times W\times 2K}$$

Where K is the number of sampling points predicted per position, and *f_offset_*denotes a 3×3 convolutional layer with 2K output channels, encoding the horizontal (x) and vertical (y) offsets for each sampling point, respectively.

A regular sampling grid *G* is constructed to cover the neighborhood around each center pixel in the feature map. The sampling positions are then obtained by adding the learned per-head offsets Δ*p* to this grid *G*. Subsequently, bilinear interpolation (BilinearSample) is performed on the feature map *F* of the other modality at these offset positions. This enables the model to flexibly extract the most relevant cross-modal information within the local receptive field. The features sampled in this way are used to form the deformed key $\tilde{K}$ and value $\tilde{V}$ for subsequent attention weight computation. For any spatial position *p* = (*x,y*) on thee feature map,e its dynamically sampled position set $G^{\prime }(p)$ is determined jointly by the predefined regular grid *G* and the offsets Δ*p*, which can be formulated according to Equations 5, 6 as follows:

(5) $$G^{\prime }(p)={\left\{p+\delta_{k}+\text{Δ}p_{k}(p)\right\}}_{k=1}^{K}$$

(6) $$\tilde{F}=\text{BilinearSample}(F,G^{\prime })$$

Where *δ_k_*denotes the predefined relative regular offset of the k-th sampling point, and Δ*p_k_*(*p*) represents the corresponding offset predicted by the network for position *p*.

Cross-modal attention in the DBCAM is computed bidirectionally, encompassing both the RGB-to-MS and MS-to-RGB pathways. In the RGB→MS pathway, the most relevant spectral-contextual information is retrieved and aggregated from the multispectral features by the query vector $Q_{rgb}$. Specifically, $Q_{rgb}$ is matched against the deformably aligned MS key features ${\tilde{K}}_{ms}$ to compute attention weights. These weights are then used to perform a weighted fusion of the MS value features ${\tilde{V}}_{ms}$, thereby enabling nitrogen-sensitive spectral responses to be injected into the RGB feature representation, which enhances its semantic awareness of nitrogen status. Conversely, in the MS→RGB pathway, $Q_{ms}$ is made to interact with the deformably sampled RGB key and value features, ${\tilde{K}}_{rgb}$ and ${\tilde{V}}_{rgb}$, respectively. Through this interaction, high-frequency texture and edge details from the RGB modality are incorporated, compensating for the inherent lack of spatial detail in MS imagery. The detailed computation of the bidirectional cross-modal attention is formulated according to Equations 7, 8 as follows:

(7) $$Attn_{rgb\to ms}=\text{softmax}(\frac{Q_{rgb}{\tilde{K}}_{ms}^{T}}{\sqrt{d}}){\tilde{V}}_{ms}$$

(8) $$Attn_{ms\to rgb}=\text{softmax}(\frac{Q_{ms}{\tilde{K}}_{rgb}^{T}}{\sqrt{d}}){\tilde{V}}_{rgb}$$

Where *softmax* performs row-wise normalization, converting attention weights into a probability distribution. The term $\sqrt{d}$ serves as a scaling factor to avoid overly large dot-product magnitudes, thereby mitigating the risk of gradient vanishing. This process enables the injection of nitrogendiscriminative spectral information into high-resolution texture representations.

The multi-head attention outputs from both pathways are concatenated along the channel dimension and further processed through a 1×1 convolution. The resulting features are then fused with the original modality representations through learnable residual weights *γ_rgb_*and *γ_ms_*, as given in Equations 9–11:

(9) $${F^{\prime }}_{rgb}=\gamma_{rgb}\cdot Attn_{rgb\to ms}+F_{rgb}$$

(10) $${F^{\prime }}_{ms}=\gamma_{ms}\cdot Attn_{ms\to rgb}+F_{ms}$$

(11) $$F_{out}={F^{\prime }}_{rgb}+{F^{\prime }}_{ms}$$

This adaptive mechanism allows the model to balance cross-modal information flow across different spatial regions effectively, preventing the introduction of redundant features in areas that are noisy or weakly correlated. Through this bidirectional attention design, the DBCAM achieves a complementary fusion between modalities, effectively coupling spectral sensitivity with spatial precision to enhance the robustness and accuracy of wheat leaf nitrogen monitoring.

#### 2.5.2 Multi-modal adaptive pyramid attention feature pyramid network

**Figure 7:**
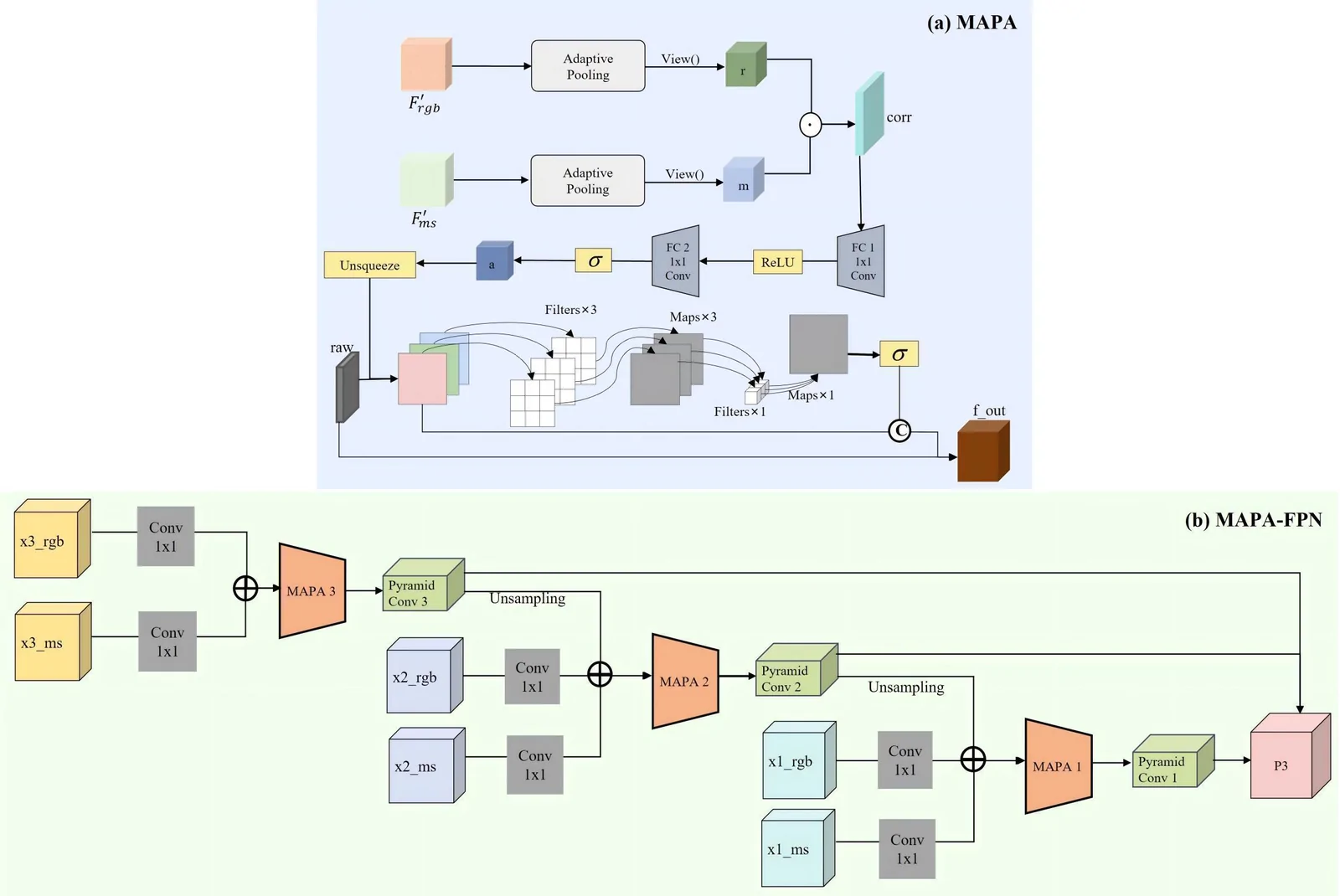
Structure of the MAPA-FPN module. **(a)** MAPA. **(b)** MAPA-FPN.

Features at various scales inherently possess rich semantic content. Although the conventional FPN promotes multi-scale fusion via a top-down pathway, it does not integrate cross-modal attention, consequently restricting the ability to capture correlations between nitrogen-sensitive spectral bands and visible features. To address this deficiency, we introduce a MAPA mechanism (**Figure 7a**) and construct a hierarchical MAPA-FPN network (**Figure 7b**). This network is designed to robustly enhance multi-scale and cross-modal feature representations under complex field conditions.

For the input features *F_rgb_*and *F_ms_*, Adaptive Average Pooling is first applied to compress the spatial dimensions into a single scalar for each feature channel. Subsequently, a view transformation is performed to extract two channel-wise statistical vectors: the RGB channel descriptor *r* and the MS channel descriptor *m*. A correlation operation (Corr) is then applied between *r* and *m* to establish semantic dependencies across modalities, thereby quantifying the intrinsic relationship between nitrogen-sensitive spectral bands and RGB features, and enabling preliminary cross-modal information fusion.

Following this, a two-layer fully connected network (Multilayer Perceptron, MLP) is employed to perform nonlinear mapping, extracting the final channel attention weights *a* from the resulting correlation map. A Sigmoid activation *σ* is applied to ensure that the attention weights are normalized within the range [0, 1], facilitating adaptive channel-wise feature selection. This process is formulated according to Equation 12 as follows:

(12) $$a=\sigma (F_{C_{2}}(\delta (F_{C_{1}}(r⊙m))))$$

Where *δ* denotes the ReLU activation function, and *σ* represents the Sigmoid activation function.

Spatial attention in the MAPA-FPN is hierarchically computed through multi-scale context aggregation and channel-modulated refinement. The input is processed by parallel 3 × 3 convolutions to capture multi-scale contexts, followed by successive 1 × 1 convolutions that transform channels and construct a feature pyramid. Channel attention weights are integrated via element-wise multiplication to enhance informative channels. Finally, a 1 × 1 convolution compresses the features into a single-channel map, with Sigmoid activation yielding the spatial attention weights s as given in Equation 13:

(13) $$s=\sigma \;({\text{Conv}}_{1\times 1}^{\text{Maps}\times 1}({\text{Conv}}_{3\times 3}^{\text{Maps}3\times 3}({\text{Conv}}_{3\times 3}^{\text{Filters}\times 3}(F_{\text{raw}})))⊙a)$$

The output features *F_out_* are computed by modulating the original input *F_raw_* with the learned spatial attention map s through element-wise weighting, and then adding the result to *F_raw_*via a residual connection, formulated in Equation 14:

(14) $$F_{out}=(F_{raw}⊗s)+F_{raw}$$

Within the MAPA-FPN framework (**Figure 7b**), the module is seamlessly integrated into a three-level feature pyramid (*P*_3_*,P*_4_*,P*_5_) to propagate high-level semantics downwards and enhance interactions across multiple feature levels. At each level, RGB and MS features are initially aligned via 1 × 1 convolution for channel consistency, then fused into intermediate features *f_i_*. After modal fusion and attention refinement in the MAPA module, enriched pyramid features *P_i_*are generated. These are upsampled and summed with corresponding lower-level multimodal features, yielding fused multi-scale representations. The final output consists of three multi-scale fused features, as given in Equations 15, 16:

(15) $$f_{i}={\text{Conv}}_{1\times 1}(x_{rgb}^{i},\;x_{ms}^{i})+\text{Upsample}(f_{i+1}),\;i=4,3$$

(16) $$P_{i}=\text{MAPA}({\text{Conv}}_{1\times 1}(x_{rgb}^{i},\;x_{ms}^{i}))+\text{Upsample}(P_{i+1}),\;i=3,4,5$$

Where $x_{rgb}^{i}$ and $x_{ms}^{i}$ denote the input RGB modal feature and MS modal feature at the i-th level, respectively. *Upsample* represents the upsampling operation, which increases the spatial resolution of higher-level features to match that of the adjacent lower level, thereby enabling spatial-level feature fusion.

At each tier, attention is derived from both the current features and upsampled refined information from higher levels, enabling a dynamic interplay between local details and global context. This facilitates efficient multimodal and multi-scale feature fusion. Through hierarchical aggregation and consistent modeling across multiple scales, MAPA-FPN successfully preserves fine details and global semantics, achieving precise and robust nitrogen-level monitoring under complex field conditions.

To assess the efficacy of the MAPA-FPN, a comparative analysis was conducted among five representative feature pyramid architectures, as detailed in **Table 3**. Within this study, the traditional FPN serves as a benchmark due to its capability for multi-scale feature fusion. The SE-FPN introduces channel-wise attention to enhance spectrally sensitive features; however, its overall performance improvement remains modest. CBAM-FPN, which simultaneously incorporates channel and spatial attention, achieves more fine-grained feature selection but still falls short in terms of mIoU and F1 scores compared to the benchmark. Conversely, A2-FPN leverages a self-attention mechanism to model global dependencies across scales, enhancing semantic consistency.

**Table 3:** Comparison experiment of optimal FPN mechanisms.

Method	mIoU(%)	Average F1(%)	Params(M)	FLOPs(G)
FPN	65.26	77.73	21.23	276.22
SE_FPN	65.13	77.64	19.93	236.71
CBAM-FPN	64.42	76.96	19.54	225.41
A2-FPN	65.27	77.41	21.48	290.32
MAPA-FPN	66.35	78.37	19.91	236.89

Differing from these architectures, the newly proposed MAPA-FPN integrates an adaptive fusion mechanism across multimodal and multi-scale levels, effectively harnessing the complementary information from both visible and MS features. This approach results in an mIoU of 66.35% and an average F1 score of 78.37%, surpassing the traditional FPN by 1.09% and 0.64% respectively. Furthermore, MAPA-FPN achieves the highest performance metrics with a relatively low param count of 19.91 million and a modest computational demand of 236.89 G FLOPs. This efficiency underscores that the cross-modal adaptive mechanism significantly enhances semantic complementarity and consistency, thereby facilitating more precise discrimination of regions with varying nitrogen levels. Overall, MAPA-FPN exemplifies optimal balance and generalization capability in the realms of multimodal feature fusion and multi-scale feature representation.

#### 2.5.3 Dual-path decoder

To address the challenges of interference from complex backgrounds, such as soil, straw, and weeds, and to diminish spectral confusion caused by blurred leaf boundaries in low-altitude UAV imagery of wheat, a dual-path decoding architecture has been developed. This architecture functions to decode background and foreground regions separately.

The background decoder adopts a progressive feature fusion strategy, gradually restoring spatial information of the image from high-level semantics to low-level details. The deepest feature output by the encoder, with a spatial resolution of 1/16 of the input image, is first upsampled to 1/2 scale via eightfold bilinear interpolation, expanding the feature map size to provide spatial support for subsequent detail fusion. Through skip connections, shallow features at the same scale from the encoder are introduced and element-wise summed with the upsampled features. This leverages the rich edge and textural details in shallow features to enhance deep features, resulting in more detailed representations and achieving an initial enhancement of spatial precision.

**Figure 8:**
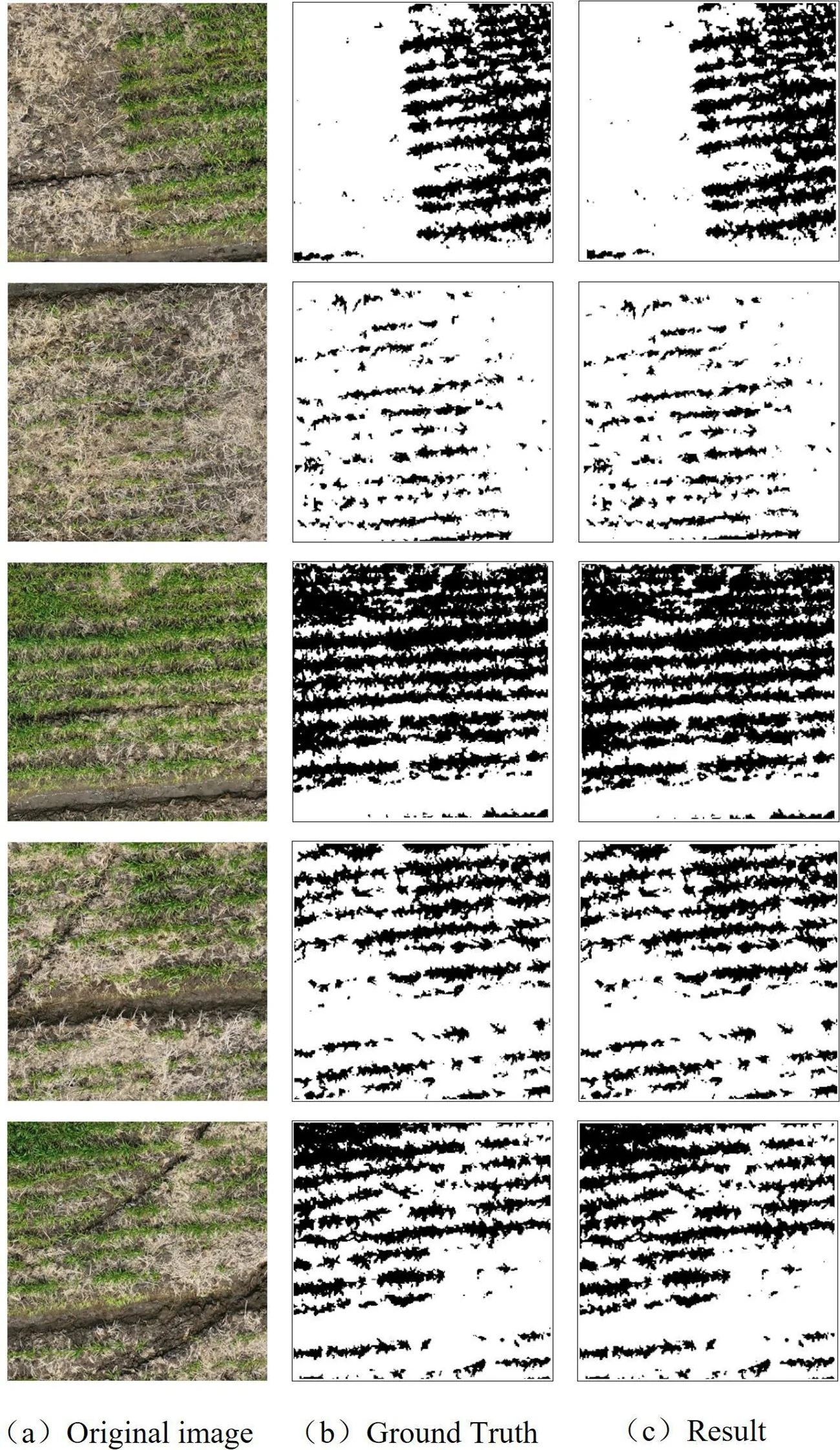
Output of the vegetation attention map. **(a)** Original RGB image. **(b)** Ground truth. **(c)** Predicted vegetation attention map.

The detail-enhanced features are then channel-wise concatenated with the 1/4-scale pyramid features from MAPA-FPN. This balances the spatial detail richness and semantic completeness at this scale, preserving the texture characteristics of wheat leaves while maintaining robust category discrimination. A 1 × 1 convolution is subsequently applied to fuse the cross-scale features from different depths and receptive fields. To further enhance the model’s perception of the unique morphology of wheat leaves, an asymmetric convolution combination is introduced for directional texture enhancement. First, a 1 × 3 horizontal convolution kernel is used to capture the lateral extensibility texture of wheat leaves, followed by a 3 × 1 vertical convolution kernel to reinforce longitudinal stripe and venation information. This approach models the slender geometric features of wheat leaves with fewer parameters. Finally, the enhanced features are mapped to a single-channel feature map via a 1 × 1 convolution, normalized to the [0,1] range using a Sigmoid activation function, and output as a continuous-probability vegetation attention map (**Figure 8**). This guides the network to focus on wheat canopy regions, effectively suppressing interference from complex backgrounds such as soil and shadows.

The foreground decoder employs a symmetric encoder-decoder structure, performing progressive feature fusion and attention modulation at each feature upsampling level. The decoder first concatenates high-resolution features from the RGB and MS encoders at the same scale with the corresponding pyramid features provided by MAPA-FPN via channel-wise concatenation, resulting in fused features.

The vegetation attention map generated by the background decoder is processed through a 3 × 3 convolution and a Sigmoid activation function to produce spatial attention weights. These weights are then element-wise multiplied with the fused features, applying weighting to the fused representation. The weighted features are subsequently upsampled and further fused with shallow features, progressively restoring spatial details level by level. Finally, the features are processed by a 1 × 1 convolution and a Softmax activation function. This design enables the foreground decoder, guided by the vegetation attention map, to more accurately distinguish wheat plants from the complex background, ultimately achieving foreground segmentation of wheat leaf nitrogen levels with clear boundaries and intact structures.

**Figure 9:**
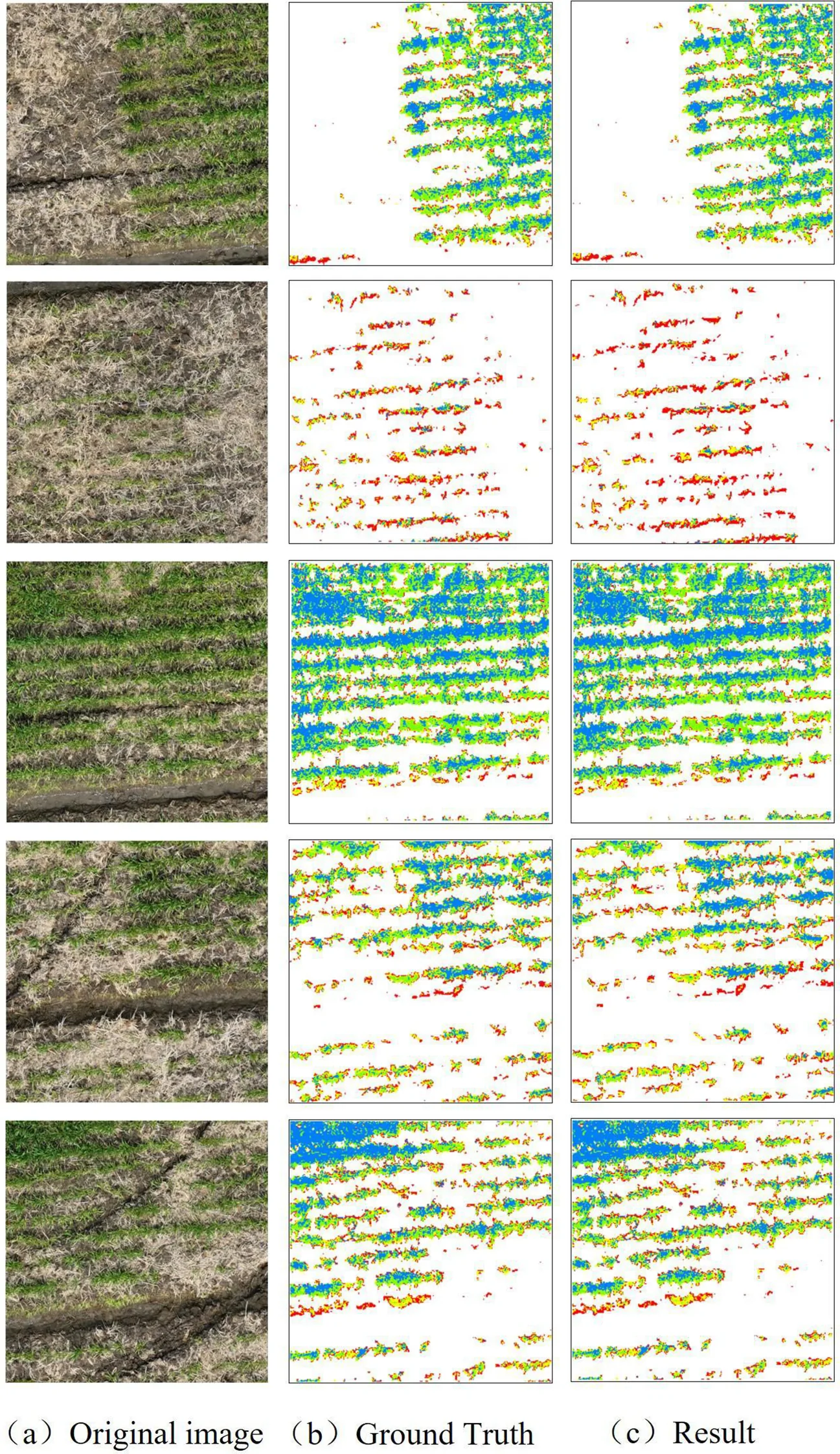
Segmentation results of wheat nitrogen levels under complex farmland backgrounds. Four pseudo-colors are used to represent different nitrogen levels: blue for nitrogen enrichment, green for optimum levels, yellow for moderate deficiency, and red for severe deficiency. **(a)** Original RGB image. **(b)** Ground truth. **(c)** Predicted segmentation results.

The monitoring outcomes for wheat leaf nitrogen levels against complex farmland backgrounds are depicted in **Figure 9** using four pseudo-colors, blue, green, yellow, and red, to respectively signify nitrogen enrichment, optimum levels, moderate nitrogen deficiency, and severe nitrogen deficiency. These colors reflect the spatial heterogeneity in nitrogen distribution. By leveraging the attention mechanism, the dual-path decoding architecture facilitates the foreground decoder in accurately delineating wheat plant areas amidst interference from soil, straw, and weeds. This capability enables high-precision leaf nitrogen segmentation and provides a reliable foundation for field monitoring and fertilization management.

## 3 Results

### 3.1 Implementation details

All models in this study were implemented and trained utilizing the PyTorch framework within the Anaconda environment, with acceleration provided by an NVIDIA RTX 4090 GPU. The batch size was established at 6 to balance GPU memory utilization and gradient estimation stability. Training was conducted over 200 epochs to ensure convergence on the validation set and effective acquisition of fine-grained features, resulting in stable segmentation performance.

### 3.2 Evaluation metrics

In this study, the IoU, F1-score, accuracy, and loss function were employed as evaluation metrics to comprehensively assess the model’s performance in wheat canopy nitrogen segmentation. The IoU is particularly effective in reflecting spatial overlap accuracy among different regions and is notably suitable for agricultural imagery, which is characterized by extensive background areas and class imbalances. The F1-score is more sensitive to small or sparsely distributed nitrogen-level regions. IoU and F1-score served as the primary evaluation metrics, while accuracy and loss were primarily used to monitor the training process and analyze model convergence.

The IoU measures the spatial overlap between the predicted results and the ground truth labels. Equation 17 gives its calculation formula as:

(17) $$IoU=\frac{TP}{TP+FP+FN}$$

The F1-score comprehensively considers both precision and recall, with its calculation defined as in Equations 18, 19:

(18) $$F1=2\times \frac{precision\times recall}{precision+recall}$$

(19) $$precision=\frac{TP}{TP+FP},\;recall=\frac{TP}{TP+FN}$$

where TP, FP, and FN represent the numbers of true positives, false positives, and false negatives, respectively.

The mean Intersection over Union (mIoU) represents the average IoU across all categories, whereas the Average F1-score (Average F1) denotes the mean F1-score for all categories. The IoU and F1-score are complementary in evaluating semantic segmentation performance: the former emphasizes the global spatial overlap, while the latter focuses on the accuracy and completeness of local predictions. Therefore, combining these two metrics provides a comprehensive and reliable assessment of model performance in the stratified nitrogen-level segmentation of the wheat canopy.

Accuracy is employed to monitor the overall convergence and optimization stability of the model. It measures the proportion of correctly predicted pixels over all pixels, and its calculation formula given in Equation 20:

(20) $$Accuracy=\frac{TP+TN}{TP+TN+FP+FN}$$

where TN denotes the number of true negatives.

Considering the characteristics of nitrogen distribution in wheat, a combined loss function comprising Tversky Loss and class-weighted Focal Loss is utilized for optimization in DMP-UNet. Tversky Loss reduces the false positive rate in minority categories or sparse regions, while the sensitivity to nitrogen-deficient areas is enhanced by adjusting the weights *α* and *β* for FP and FN. The class-weighted Focal Loss improves the model’s ability to discriminate hard-to-classify samples, particularly in boundary transition regions. The core formulation of this approach is expressed according to Equations 21–23 as follows:

(21) $$L_{\text{Tversky}}=1-\frac{TP}{TP+\alpha \cdot FP+\beta \cdot FN}$$

(22) $$L_{\text{Focal}}=-w_{\text{c}}(1-p_{\text{c}}{)}^{\gamma }\text{log }(p_{\text{c}})$$

The combined loss function is defined as:

(23) $$L_{Combined}=(\begin{array}{c} 1-\lambda \end{array})L_{Focal}+\lambda L_{Tversky}$$

where *λ* controls the relative weight of the two loss components, *w_c_*denotes the class weight, and *γ* is the focusing parameter of the Focal Loss. Both global regional consistency and local boundary accuracy are considered in this design, enabling the model to be substantially superior to the baseline in terms of IoU and F1 scores.

In contrast, a linear combination of Focal Loss and Dice Loss is typically employed by commonly used contrast models, as given in Equations 24, 25:

(24) $$L_{Dice}=1-\frac{2\cdot (pred\cdot target)}{pred+target}$$

(25) $$L_{Combined}^{baseline}=\lambda L_{Focal}+(1-\lambda )L_{Dice}$$

In DMP-UNet, global regional consistency and local boundary accuracy are better balanced through category weighting and adjustable loss weights, allowing for improved performance on sparse targets and in complex backgrounds.

### 3.3 Analysis of modality contribution

To assess the contribution of each modality in the proposed multimodal framework, an ablation study was carried out with the following input settings: RGB-only, MS-only, and fused RGB+MS. The quantitative results are summarized in **Table 4**. The RGB-only model yields competitive performance, which can be attributed to its strong spatial and texture representation capabilities. In contrast, the MS-only model supplies complementary spectral information relevant to nitrogen sensitivity, though its performance is constrained by insufficient spatial detail. By integrating RGB and MS modalities, the proposed model achieves a significant boost in both mIoU and F1-score. Compared to single-modality baselines, the multimodal fusion enhances robustness in complex backgrounds and reduces confusion between similar nitrogen levels.

**Table 4:** Performance of different modalities.

Modality	mIoU(%)	Average F1(%)
RGB-only	49.98	65.02
MS-only	61.09	73.75
RGB+MS	66.35	78.37

**Figure 10:**
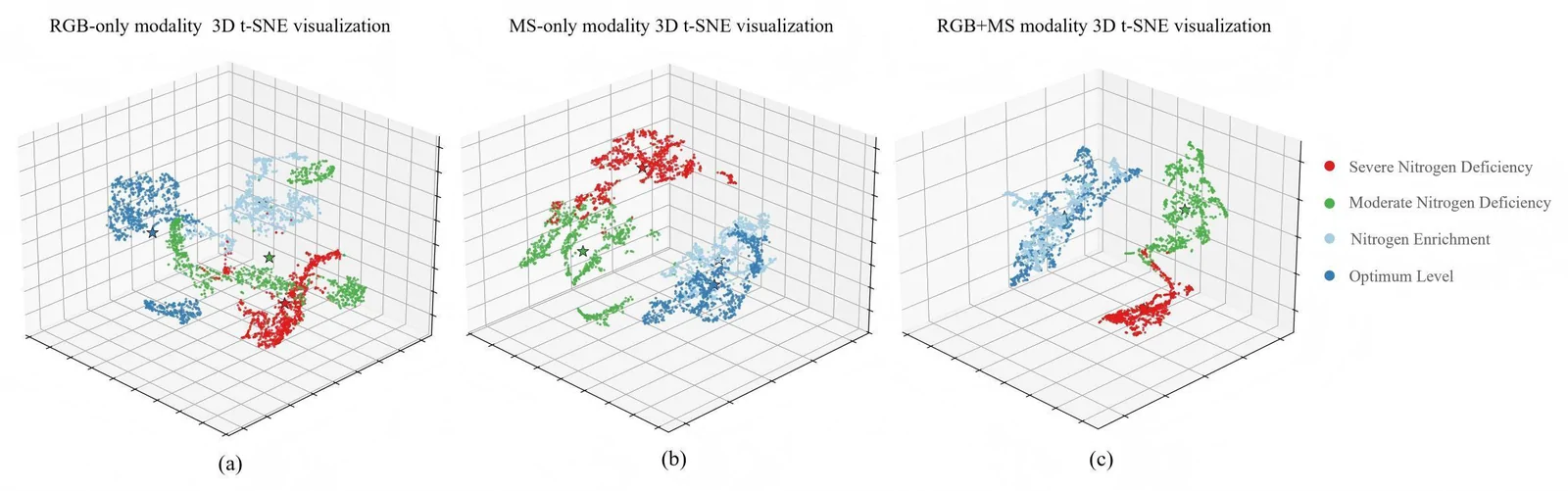
3D t-SNE visualization of different input modalities. **(a)** RGB-only. **(b)** MS-only. **(c)** RGB+MS multimodal.

A 3D t-distributed stochastic neighbor embedding (t-SNE) visualization of three input modalities, RGB-only, MS-only, and fused RGB+MS, is presented in **Figure 10**. The visualization shows that features from the fused RGB+MS modality form more distinct and compact clusters compared with the single-modality inputs, indicating improved separation among different wheat nitrogen levels.

For the RGB-only modality (**Figure 10a**), features corresponding to the four nitrogen levels (Severe Nitrogen Deficiency, Moderate Nitrogen Deficiency, Optimum Level, and Nitrogen Enrichment) exhibited severe overlap without distinct inter-class boundaries. This can be attributed to the fact that RGB imagery, while capturing rich canopy texture and morphological details, lacks sufficient spectral sensitivity to subtle variations in wheat nitrogen content, resulting in poor discriminability for fine-grained nitrogen status differentiation. For the MS-only modality (**Figure 10b**), partial separation of feature clusters was observed across nitrogen levels, particularly for the Severe Nitrogen Deficiency class, which formed a relatively independent aggregation. Nevertheless, notable feature overlap persisted between the Optimum Level and Nitrogen Enrichment groups, as well as between Moderate and Severe Nitrogen Deficiency. This demonstrates that singlemodal multispectral data, despite encoding nitrogen-sensitive spectral information, fails to fully characterize the spatial structural differences of wheat canopies under different nitrogen treatments, thus restricting the inter-class separability of extracted features. In stark contrast, the fused RGB+MS modality (**Figure 10c**) delivered exceptional clustering performance: features of each nitrogen level formed compact, highly independent clusters with clear inter-class boundaries and negligible cross-group overlap.

This visualization demonstrates that the dual-branch encoder effectively captures and fuses complementary information from the RGB and MS modalities, with spatial texture from the RGB branch and nitrogen-sensitive spectral features from the MS branch, through the proposed DBCAM and MAPA-FPN modules, substantially enhancing the discriminative power of the deep features for wheat nitrogen status classification.

### 3.4 Ablation experiment

Ablation experiments were conducted on the DMP-UNet network using the UAV wheat nitrogen image dataset to evaluate the effectiveness of the DBCAM, MAPA-FPN, and dual-path decoder modules, as well as to determine their contributions to the model’s segmentation performance, as shown in **Table 5**. The findings demonstrate that each of the three modules significantly enhances segmentation performance.

**Table 5:** Results on ablation experiments of different modules.

Method	Modules			IoU(%)					Evaluation index	
	DBCAM	DBCAM-FPN	Dual-path decoder	Severe nitrogen deficiency	Moderate nitrogen deficiency	Optimum level	Nitrogen enrichment	Back-ground	mIoU (%)	Average F1(%)
Dual_U				44.15	45.75	33.92	42.13	91.80	51.55	65.85
Dual_U+DBCAM	✓			44.91	46.28	35.37	45.97	92.21	52.95	67.22
Dual_U+MAPA-FPN		✓		61.66	57.38	42.46	45.53	96.84	60.77	73.65
Dual_U+Dual-path Decoder			✓	64.30	58.90	47.16	53.13	95.03	63.70	76.37
Dual_U+DBCAM+MAPA-FPN	✓	✓		62.97	58.00	45.66	48.37	96.80	62.36	75.11
Dual_U+DBCAM+Dual-path Decoder	✓		✓	64.76	59.90	49.90	57.15	94.56	65.25	77.74
Dual_U+MAPA-FPN+Dual-path Decoder		✓	✓	66.09	60.09	50.39	56.70	98.38	66.33	78.37
Dual_U+DBCAM+MAPA-FPN+Dual-path Decoder	✓	✓	✓	66.25	60.95	49.71	56.36	98.49	66.35	78.37

Specifically, the basic network configuration, referred to as *Dual_U_*, achieved a mIoU of 51.55% and an average F1-Score of 65.85%. With the incorporation of the DBCAM module, there was an increase of 1.40% in mIoU and 1.37% in average F1-Score, suggesting that this module facilitates the enhanced representation of key cross-modal features and achieves adaptive fusion of RGB and MS features through a dynamic sampling attention mechanism.

Building on this, the addition of the MAPA-FPN module led to further increases in mIoU by 9.41% and in average F1-Score by 7.89%, respectively. These improvements indicate that the integration of channel attention, spatial attention, and multi-scale feature fusion significantly contributes to the enhanced utilization of multimodal information.

By integrating the dual-path decoder, there was an additional increase in mIoU by 3.99% and in average F1 by 3.26%, demonstrating that this module effectively achieves background suppression and fine-grained segmentation through the foreground decoder.

From the perspective of category-wise segmentation metrics, the IoU showed significant improvement across various nitrogen levels, severe deficiency, moderate deficiency, optimum, and enriched, attributed to the synergistic application of the three modules. This comprehensive improvement underscores the effectiveness and robustness of the DBCAM, MAPA-FPN, and dual-path decoder in facilitating precise nitrogen-level segmentation in wheat.

### 3.5 Comparison with other methods

To rigorously evaluate the efficacy of DMP-UNet in the nitrogen-level segmentation of the wheat, this study conducted a comprehensive comparative analysis against established segmentation models within various representative scenarios. For this purpose, five distinct distribution patterns characterized by unique features were selected, representing typical scenarios including semi-sparse and semi-dense, sparse, dense, unevenly distributed, and complex backgrounds. This selection facilitated a thorough assessment of the model’s adaptability and robustness across diverse practical conditions. The comparison involved several models, including U-Net (Ronneberger et al., 2015), U-Net++ (Zhou et al., 2018), and U-Net 3+ (Huang et al., 2020), all of which employ classic encoder-decoder structures. These models served as benchmarks to gauge the enhancements DMP-UNet introduces over conventional segmentation architectures. Additionally, models such as Attention U-Net (Oktay et al., 2018) and Attention R2U-Net (Alom et al., 2018), which integrate attention mechanisms, were utilized to evaluate improvements in the model’s ability to concentrate on target regions amidst complex backgrounds. Furthermore, R2U-Net (Alom et al., 2018), which merges residual connections with recurrent convolutions, was used to explore the benefits of profound feature representation and multi-scale information fusion. Advanced semantic segmentation networks like DeepLabV3 (Chen et al., 2017) and DeepLabV3+ (Chen et al., 2018), incorporating dilated convolutions and ASPP, were chosen to contrast capabilities in multi-scale feature capture and boundary refinement. These models were categorized into four architectural groups: classical structures, attention-enhanced models, deep residual recurrent networks, and advanced semantic segmentation frameworks, thereby showcasing the superior performance of DMP-UNet in horizontal nitrogen-level segmentation of wheat under complex agricultural backgrounds.

**Figure 11:**
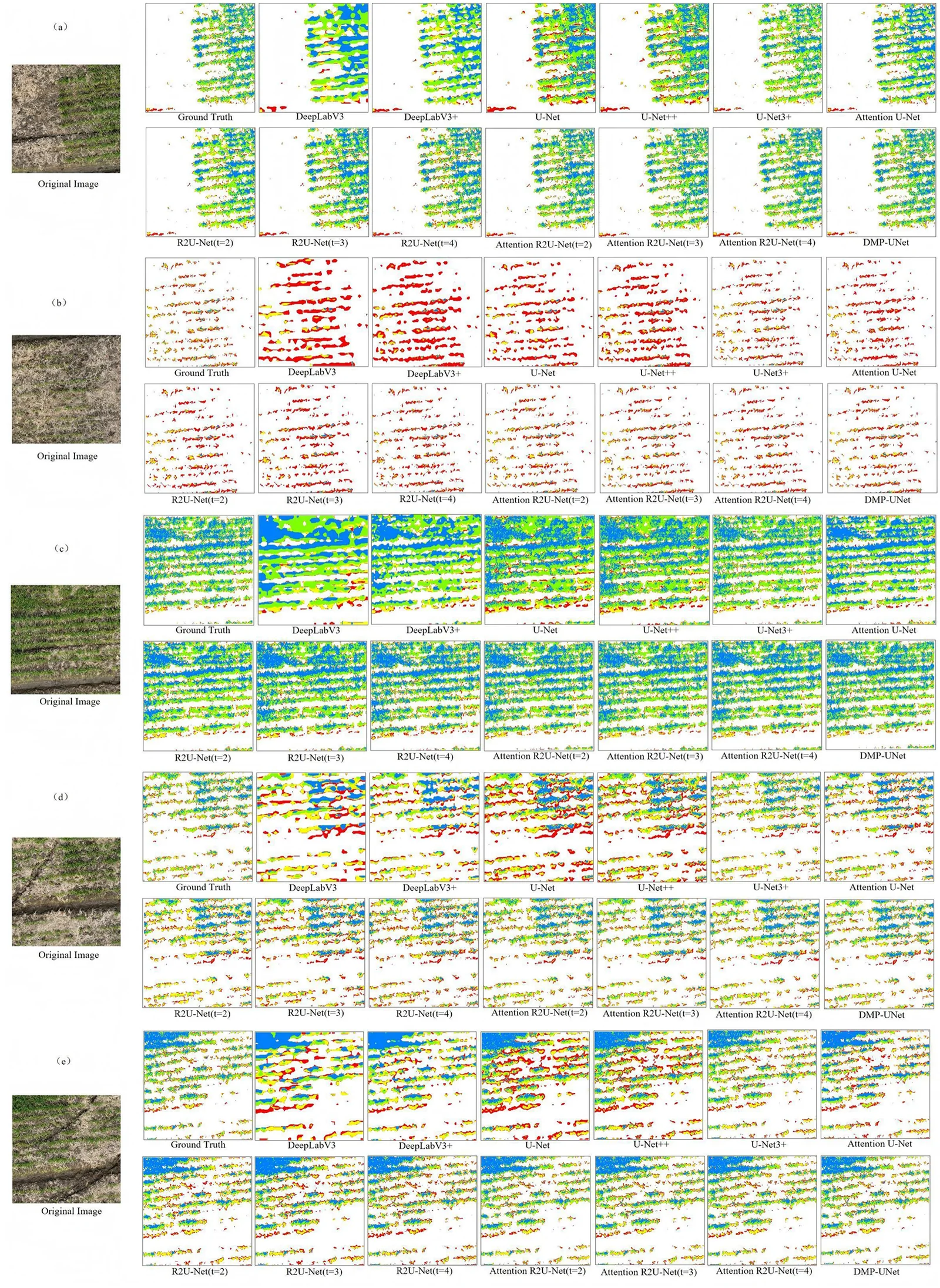
Segmentation performance of comparison models for wheat nitrogen levels under five representative scenarios. **(a)** Semi-sparse and semi-dense. **(b)** Sparse. **(c)** Dense. **(d)** Unevenly distributed. **(e)** Complex background.

As depicted in **Figure 11**, while conventional segmentation models such as DeepLabV3 and U-Net, along with their derivatives, can identify main nitrogen distribution areas in certain conditions, they generally exhibit issues such as blurred boundaries, excessive noise interference, and a lack of finegrained region representation. In contrast, DMP-UNet exhibits enhanced segmentation capabilities across various scenarios, attributed to the incorporation of cross-modal feature interaction, multiscale pyramid fusion, and dual-path decoders. This integration ensures better preservation of texture details between wheat rows, reduced impact of background interference on predictions, and consistently high segmentation accuracy and coherence, even under dense and unevenly distributed cell conditions.

The experimental outcomes (**Table 6**) demonstrate that DMP-UNet significantly surpasses the comparative models in critical evaluation metrics, with increases in mean IoU ranging from 3.46% to 32.94%, and improvements in mean F1 score from 2.89% to 33.71%. Although the performance differences among the models are relatively minor in the background category, DMP-UNet still maintains the highest performance, showing an improvement of 1.29% over the next best model.

**Table 6:** Results of comparative experiments.

Model name	IoU(%)					Evaluation index	
	Severe nitrogen deficiency	Moderate nitrogen deficiency	Optimum Level	Nitrogen enrichment	Background	mIoU(%)	Average F1(%)
DeepLabV3	29.66	27.07	16.72	11.88	79.35	32.94	44.66
DeepLabV3+	33.08	31.79	21.82	18.03	85.91	38.13	50.59
U-Net	58.83	53.70	39.93	41.75	94.81	57.80	71.08
U-Net++	59.69	55.17	40.87	43.04	95.56	58.87	72.02
U-Net3+	60.95	57.02	44.25	47.57	96.01	61.16	74.04
Attention U-Net	60.00	56.04	41.08	43.81	95.29	59.24	72.32
R2U-Net (t=2)	56.77	56.80	45.89	47.87	97.06	60.88	73.77
R2U-Net (t=3)	58.17	56.08	45.26	43.67	97.06	60.05	72.88
R2U-Net (t=4)	60.46	57.44	45.30	46.97	97.02	61.44	74.16
Attention R2U-Net (t=2)	61.37	54.68	46.55	50.25	97.32	62.03	74.72
Attention R2U-Net (t=3)	61.57	58.21	46.78	50.68	97.20	62.89	75.48
Attention R2U-Net (t=4)	61.93	57.78	45.88	51.49	97.21	62.86	75.44
DMP-UNet	66.25	60.95	49.71	56.36	98.49	66.35	78.37

A detailed comparison reveals that DMP-UNet outperforms the suboptimal model Attention R2U-Net (t=3) by achieving an increase of 3.36% in mIoU and 2.89% in average F1 score, thereby underscoring its superior overall capabilities. Category-specific analysis further demonstrates that DMP-UNet consistently delivers substantial and significant enhancements in performance across all four nitrogen level categories, severe nitrogen deficiency, moderate nitrogen deficiency, optimum level, and nitrogen enrichment, with respective mIoU improvements of 4.68%, 2.74%, 2.93%, and 5.68% compared to the suboptimal model. These results clearly indicate the model’s robust capability for fine-grained differentiation among various nitrogen levels.

In summary, DMP-UNet excels in overall performance, category discrimination, and metric consistency when compared to other models, convincingly illustrating that the proposed method enhances the accuracy and efficiency of segmenting wheat nitrogen levels in complex agricultural environments.

**Figure 12:**
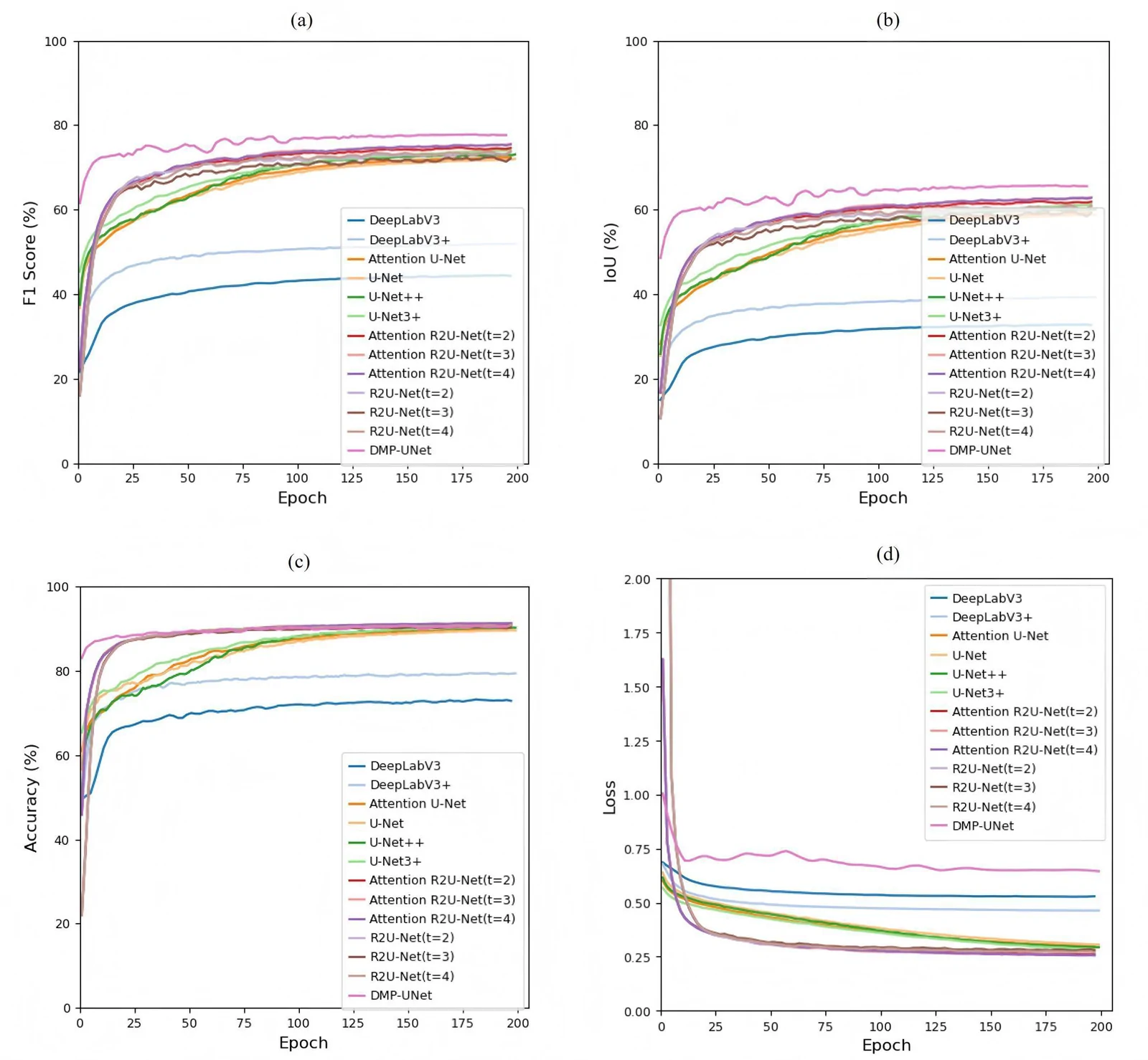
Comparison of performance curves among different models: **(a)** F1 Score, **(b)** IoU, **(c)** accuracy, and **(d)** loss.

A thorough evaluation of DMP-UNet’s segmentation performance was conducted using metrics such as average F1 score, mIoU, accuracy, and loss. The findings, depicted in **Figure 12**, show that among the models evaluated, the DeepLabV3 series recorded the lowest average F1 score, accompanied by notable false positives and false negatives. In contrast, U-Net3+ and Attention R2U-Net (t=4) reached an average F1 score of approximately 75%. Markedly, DMP-UNet consistently demonstrated the highest performance, achieving an average F1 score of 78.37% and an mIoU of 66.35%. These metrics highlight its strengths in precise target localization, detail retention, and boundary delineation, as shown in **Figures 12a, b**. Additionally, the model quickly reached an accuracy rate of approximately 90% within the initial 50 epochs and maintained this level throughout the training process, indicating rapid convergence and stable optimization (**Figure 12c**).

It is important to note that although a marginally higher training loss is observed for DMP-UNet compared with other models (**Figure 12d**), it demonstrates superior performance in the final segmentation metrics, such as mIoU, average F1 score, and Accuracy. This indicates enhanced generalization and robustness. This phenomenon may be attributed to the inherent discrepancies between the optimization objectives of the loss function and the evaluation metrics used. Specifically, DMP-UNet utilizes a combination of Tversky Loss and class-weighted Focal Loss to emphasize the segmentation accuracy of sparse foreground and boundary regions, thereby enhancing the identification of minority-category targets. However, this approach increases the sensitivity of the loss function to misclassifications in extensive background areas, leading to a marginally elevated overall loss value despite improved segmentation quality.

In contrast, the comparison models employ a combination of Focal Loss and Dice Loss, which are more forgiving of background errors and thus result in lower overall loss values. Nevertheless, these models exhibit limited capability in segmenting sparse regions and detailing boundaries, which results in inferior mIoU and average F1 scores compared to DMP-UNet. Consequently, the magnitude of the loss value does not necessarily correlate with the final segmentation performance. DMP-UNet places greater emphasis on foreground accuracy and boundary refinement, which accounts for its superior mIoU and average F1 performance relative to the baseline models. These results further demonstrate that the loss function, designed to align with the nitrogen distribution characteristics in wheat, effectively enhances the model’s fine-grained segmentation capability under complex farmland conditions.

To evaluate the reliability and generalization of DMP-UNet, a stratified ten-fold cross-validation (F1-F10) was performed(**Table 7**). The model achieved an mIoU of 63.43 ± 1.44% and an Average F1-score of 76.03 ± 1.51% across the folds. These values are slightly lower than the single-experiment results (mIoU 66.35%, Average F1 78.37%), which is reasonable, as cross-validation provides a more conservative and robust assessment over multiple independent data splits.

**Table 7:** Ten-fold cross-validation results of DMP-UNet.

Metric	F1	F2	F3	F4	F5	F6	F7	F8	F9	F10	Mean ± Std
mIoU(%)	62.03	64.85	65.97	63.71	64.22	61.63	62.14	61.95	64.01	63.82	63.43 ± 1.44(%)
Average F1(%)	74.60	77.52	78.73	76.36	76.98	74.18	74.65	74.49	76.30	76.49	76.03 ± 1.51(%)

The relatively small standard deviations indicate consistent performance across different folds. Importantly, DMP-UNet reliably distinguishes between different wheat nitrogen levels and captures their spatial distribution, confirming that the strong segmentation observed in the single experiment is not coincidental but reflects stable generalization. These results demonstrate the model’s suitability for practical field-scale precision nitrogen monitoring.

### 3.6 Cross-site generalization experiment for nitrogen level segmentation

**Figure 13:**
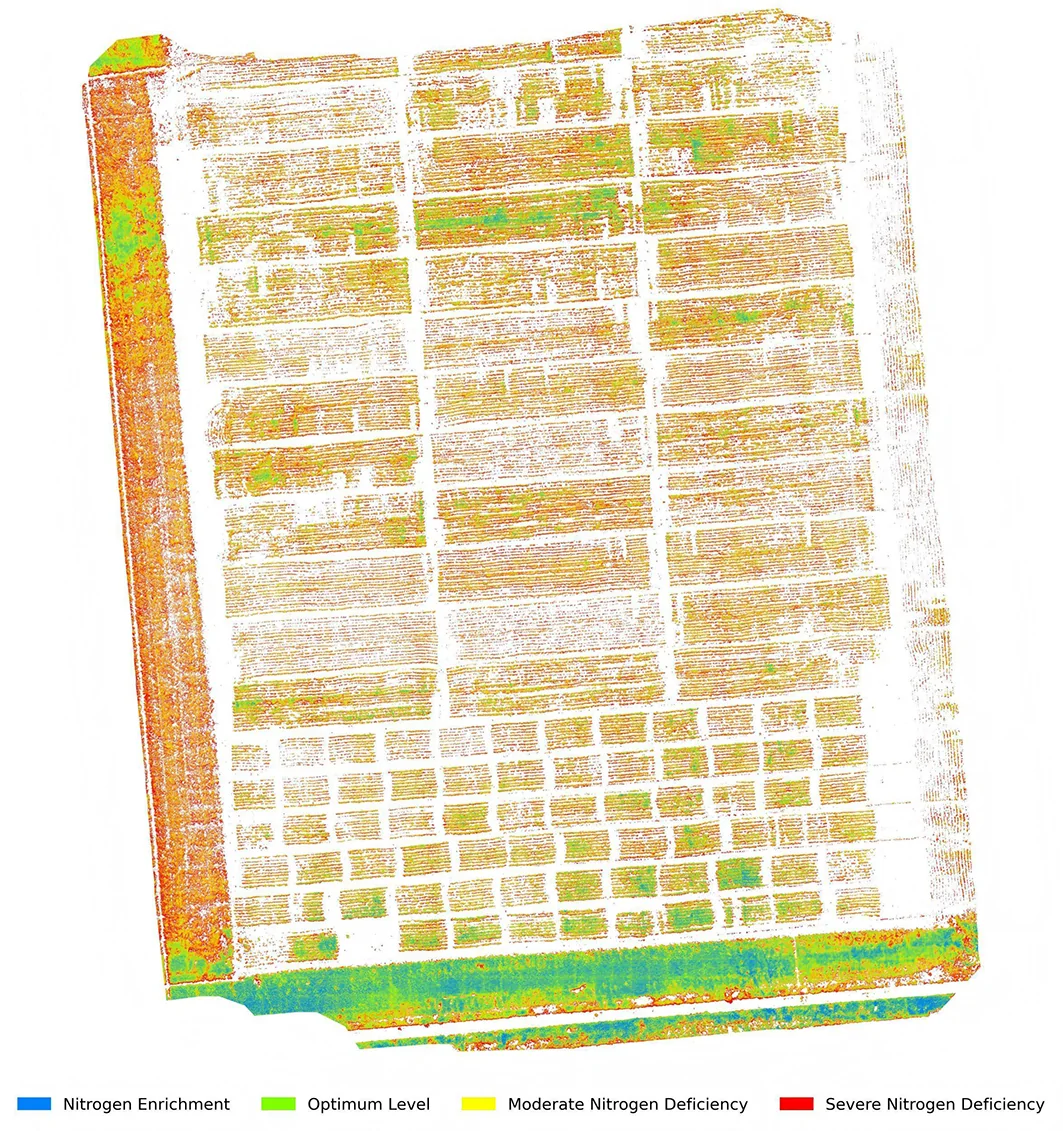
Visualization results of wheat nitrogen level segmentation in experimental Site A.

To evaluate the reliability and generalization of the proposed model for wheat nitrogen content segmentation, the visualization results obtained from the main experimental Site A (containing Zone A and Zone B) is presented in **Figure 13**. On this basis, an additional experimental Site B planted with ‘Zhen Mai 12’ was selected, which exhibits significant differences from Site A in geographical and environmental conditions, with corresponding images acquired in March 2025.

Based on this configuration, a direct cross-site validation scheme was established, in which models trained on Site A were directly evaluated on Site B. To objectively verify the generalization capacity of the proposed model, all comparative methods were tested on Site B to qualitatively benchmark their cross-site generalization performance. The evaluation was supported by qualitative visual analysis focusing on spatial consistency, noise suppression, and semantic rationality. These experiments were designed to comprehensively validate the superiority and generalization robustness of the proposed method through comprehensive model comparisons.

**Figure 14:**
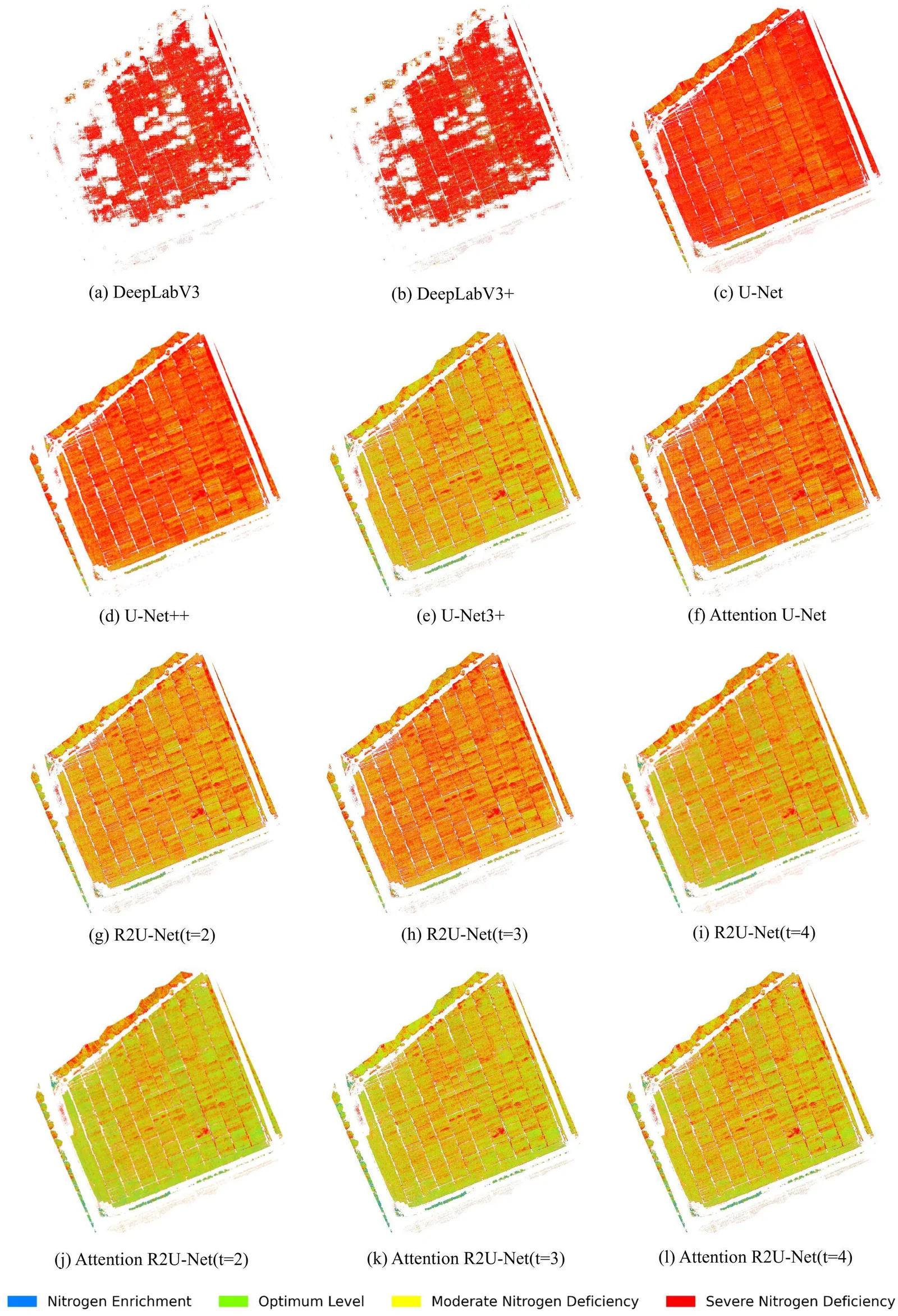
Visual illustration of wheat nitrogen level segmentation patterns across all comparative models for site B. **(a)** DeepLabV3; **(b)** DeepLabV3+; **(c)** U‑Net; **(d)** U‑Net++; **(e)** U‑Net3+; **(f)** Attention U‑Net; **(g)** R2U‑Net (t=2); **(h)** R2U‑Net (t=3); **(i)** R2U‑Net (t=4); **(j)** Attention R2U‑Net (t=2); **(k)** Attention R2U‑Net (t=3); **(l)** Attention R2U‑Net (t=4).

Visual illustration of wheat nitrogen level segmentation patterns across all comparative models for Experimental Site B is presented in **Figure 14**. DeepLabV3 and DeepLabV3+ were primarily characterized by inaccurate segmentation of field boundaries. Predictions from U-Net and U-Net++ were dominated by severe nitrogen deficiency, with the fine-grained spatial variations of nitrogen content within the field largely overlooked. Pronounced noise and misclassifications were observed in the outputs of Attention U-Net and R2U-Net (t=2, 3), particularly in the transition zones between severe nitrogen deficiency and the optimum level. Smoother and more continuous nitrogen distribution maps that better reflect real field conditions were generated by U-Net3+, R2U-Net (t=4), and Attention R2U-Net (t=2, 3, 4). Side-by-side visual comparisons intuitively reflect the cross-site adaptability of the proposed DMP-UNet relative to other competing segmentation architectures, which demonstrates its favorable suitability for precise wheat nitrogen nutrition monitoring using UAV remote sensing imagery.

**Figure 15:**
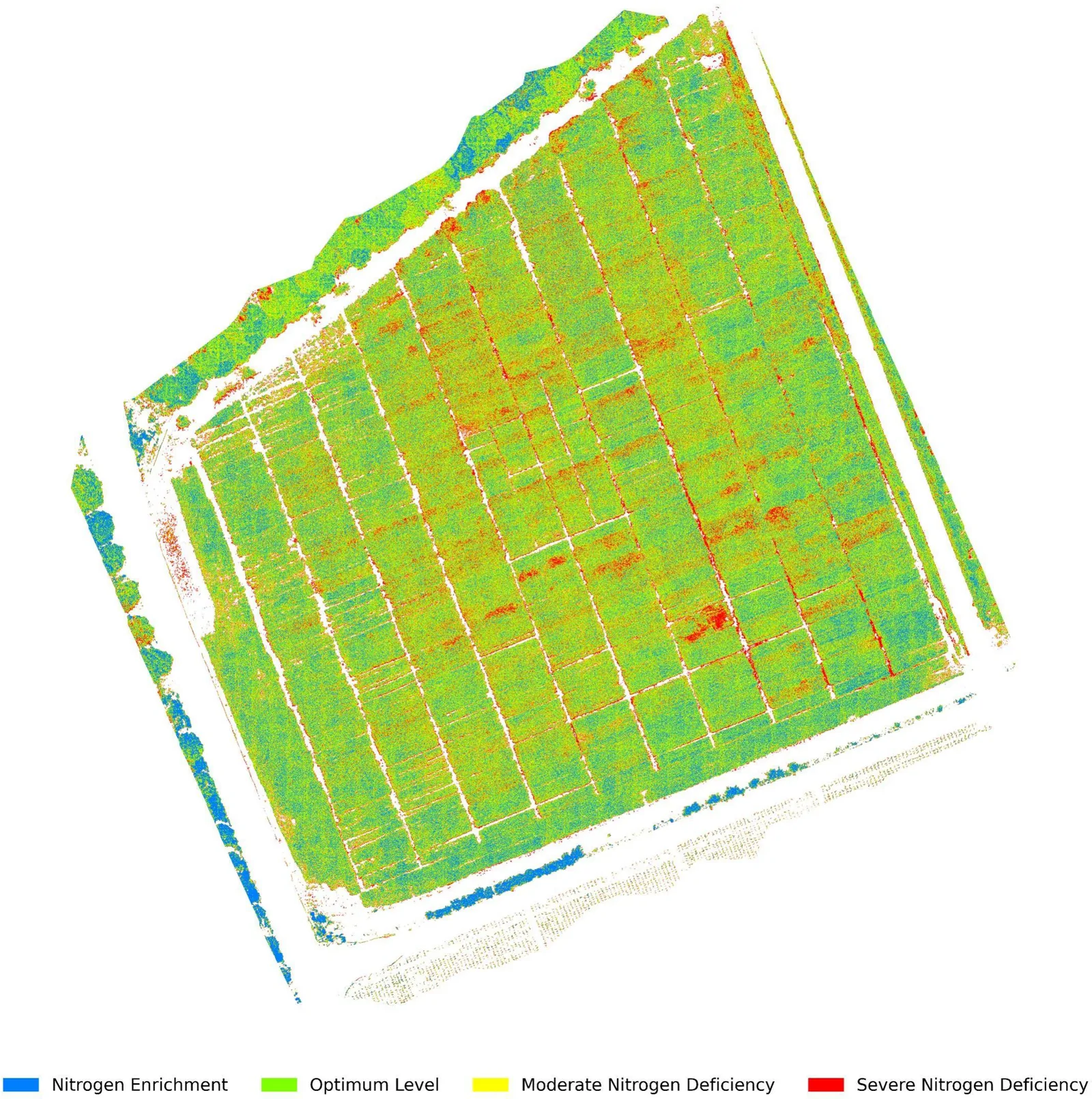
Visual illustration of wheat nitrogen level segmentation patterns for experimental site B.

Visual illustration of wheat nitrogen level segmentation patterns for Experimental Site B is presented in **Figure 15**, where progressive refinement of segmentation is achieved, enabling the gradient from severe nitrogen deficiency to the optimum level to be captured more accurately with fewer classification errors.

## 4 Conclusions

This study introduces DMP-UNet, an innovative network model optimized for nitrogen-level segmentation of wheat canopies against complex backgrounds. The model incorporates a dual-path encoder architecture to independently process RGB and MS data, facilitating modality-specific feature extraction. A DBCAM is employed to dynamically align and deeply fuse multimodal features, thus capitalizing on the complementary nature of visible and spectral information. Additionally, the MAPA-FPN is crafted to effectively assimilate multi-scale contextual data while integrating both channel and spatial attention mechanisms. This architecture significantly boosts the model’s capacity to manage scale variations and intricate background interference. In the decoding phase, a dual-path decoder is utilized, distinctly segregating background and foreground processing. The generation of a vegetation attention map directs the foreground decoder, enabling precise targeting of the wheat canopy region and effectively diminishing the impact of complex background elements such as soil, shadows, weeds, and extraneous noise. A series of incremental ablation experiments were conducted to verify the independent effectiveness of each core component. The MAPA-FPN and dual-path decoder bring prominent performance improvements under fixed loss function settings, while DBCAM delivers obvious cross-modal feature alignment gains on the basic dual-encoder structure and enhances the cross-site generalization ability of the complete model.

Experimental evaluations performed on a self-constructed multimodal wheat canopy dataset affirm that DMP-UNet proficiently accomplishes multimodal information fusion. The dataset was built based on sparse field destructive sampling, where pixel-wise pseudo-labels were extrapolated from Random Forest using measured nitrogen data, matching the practical sampling constraints of precision agriculture. By maintaining detailed structural integrity, the model markedly improves the precision and robustness of nitrogen-level segmentation in wheat. When compared with prevailing mainstream segmentation models, DMP-UNet registers significant enhancements in key evaluation metrics, including IoU, accuracy, and F1 score. Further cross-site qualitative visual comparisons on an independent experimental area visually verify the outstanding cross-region adaptability of DMPUNet against competing segmentation architectures, which can reliably capture continuous nitrogen gradient changes from severe deficiency to optimum nitrogen status with fewer classification errors. This model demonstrates superior efficacy in challenging conditions, such as sparse canopies and environments with potent background interference, underscoring its considerable potential for application in practical agricultural settings. However, opportunities for enhancement in detail restoration and segmentation accuracy persist.

Future research will concentrate on the further refinement of the network architecture, encompassing the incorporation of more sophisticated feature reconstruction mechanisms, improved representation of small-target and edge features, and the integration of adversarial training strategies to augment the model’s generalization capabilities under complex conditions. Comprehensive validation will be pursued on expanded and multi-growth-cycle wheat MS datasets. We will also explore weakly-supervised and self-supervised learning frameworks to reduce reliance on RF-generated pseudo-labels, and adopt spatially independent plot-level dataset partitioning strategies for more rigorous generalization testing. Additionally, efforts will be made to merge this technology with established agricultural knowledge to forge more interpretable and dependable nitrogen diagnosis paradigms. These initiatives are directed towards furnishing stable, robust, and practical deep learning solutions for accurate nitrogen monitoring and intelligent decision-making in the field of agricultural remote sensing.

## Data Availability

The original contributions presented in the study are included in the article/supplementary material. Further inquiries can be directed to the corresponding author.
